# Environmental risk assessment of the Nile Delta, Egypt, based on radar interferometry, altimetry, and geodetic measurements

**DOI:** 10.1038/s41598-025-03831-w

**Published:** 2025-06-01

**Authors:** Soha Hassan, Mohamed Saleh, Bayoumy Mohamed, Mohamed S. Elhebiry, Abdelaziz Abdeldayem, Elsayed Issawy, Khaled Zahran, Samir Kamh

**Affiliations:** 1 Department of Geodynamics, National Research Institute of Astronomy and Geophysics (NRIAG), Helwan, 11421 Egypt; 2 GeoHydrodynamics and Environment Research (GHER), University of Liege, Liege, Belgium; 3 Oceanography Department, Faculty of Science, Alexandria University, Alexandria, 21500 Egypt; 4 Department of Geology, Al-Azhar University, Cairo, 11884 Egypt; 5 Earth and Environment Department, Albion College, Albion, MI49224 USA; 6 Department of Geology, Faculty of Science, Tanta University, Tanta, 31527 Egypt

**Keywords:** Nile Delta, Sentinel-1, GNSS, Altimetry, Tide gauges, Land subsidence, SLR, inundation, simulation model, Environmental sciences, Environmental impact, Geodynamics

## Abstract

Egypt is confronted with a number of hazardous environmental incidents, mainly sea level rise (SLR) and land subsidence. The Nile Delta is a low-relief surface that is particularly vulnerable to flooding and SLR, making it important to study inundation scenarios for the region. Potential social and economic consequences of this anticipated sea encroachment were projected utilizing (1) crustal deformation calculations derived from the time series analysis using the Persistent Scatterer Interferometry (PSI) technique based on Least Squares Estimation. (a stack of 191 Sentinel-1 ascending scenes), and eight permanent stations of Global Navigation Satellite System (GNSS); both spanning the period 2014–2019, (2) SLR values using Satellite Altimetry, and (3) a high-resolution digital elevation model (TerraSAR-X/TanDEM-X). The The key findings of this study are summarized as follows; (1) large cities and urban regions adjacent to the two main active branches of the Nile Delta (Rosetta and Damietta) experienced the majority of subsidence rates, (2) the cities of Damietta, Mansoura and Port said (eastern side of the Nile Delta) experienced the maximum rates of subsidence (− 11 ± 0.6, − 8.9 ± 0.7, and − 6.3 ± 0.7 mm/year, respectively), (3) the cities of Shebin El Kom, Damanhour, Tanta, New-Damietta, Kafr El-Sheikh had moderate subsidence rates (− 3.2 ± 0.6, − 2.4 ± 0.7, − 4.2 ± 0.6, − 3.8 ± 0.7, − 3.2 ± 0.7 mm/year, respectively), (4) the Nile Delta subsidence seems to be dominated by anthropogenic reasons such as urbanization, ground water and hydrocarbon extraction, (5) the linear trend of sea level anomaly (SLA) from satellite altimetry data over the period from 1993 to 2019 along the Delta shoreline, the SLR is ~ 3.42 ± 0.5 mm/year, and (6) based on GIS tools and IDW interpolation, wide swaths of the northern Nile Delta would be flooded in the worst-case scenario, which would result in approximately 482 km^2^ being flooded in fifty years, 2433 km^2^ in one hundred years, and 3320 km^2^ in one hundred and fifty years due to the ongoing land subsidence and SLR of 3.4 mm/year.

## Introduction

Climate change has become a global reality, with sea-level rise (SLR) being a direct consequence of warming temperatures^1^. Thermal expansion of seawater and glacial melting are primary drivers^2–6^, while regional, local, and global factors, such as mid-ocean ridge activity^2–6^, oceanographic dynamics^2–6^, and vertical land movements^2–6^, also contribute to relative sea-level changes.

**Fig. 1:**
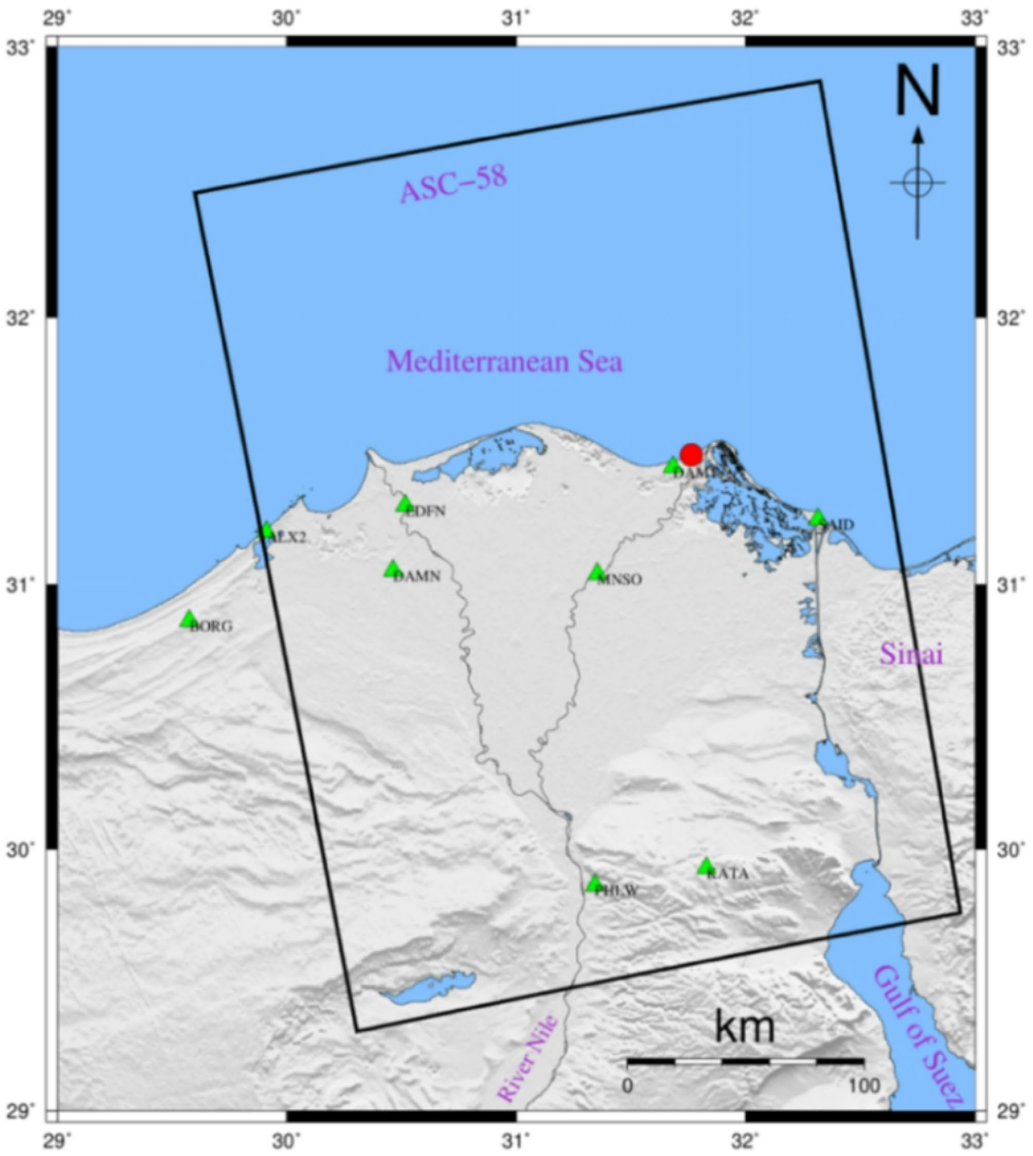
Nile Delta and surroundings basemap, showing the footprints of ascending satellite track (T58-A), with spatial distribution of the GPS stations utilized in this work (green triangles), and the location of tide gauge station in New-Damietta city (red circle). Thae map was created using Generic Mapping Tools (GMT), version 5^19^.

Globally, coastal low-lying land regions and their inhabitants are facing danger from SLR. Deltas are a prototype for such low-relief topography with a dense population. Like other deltas worldwide, the Nile Delta (Fig. 1) is considered among the most vulnerable regions to SLR^7–9^. The Nile Delta plays a crucial role in the evolution of Egyptian society and plays an intrinsic part in the country’s culture and economy. It is host for more than 42% of Egypt’s population^10^, 60% of industrial and commercial operations^11^, and more than 50% of cultivation^12^. Furthermore, the Nile Delta (offshore and onshore) is one of Northern Africa’s most potential sites for hydrocarbon production, exploration, and supply^13–15^. SLR may have negative consequences on the delta, such as increasing coastal erosion, breaching of coastal barriers, retreating of barrier dunes, increasing flooding, saltwater intrusions into delta aquifer, destruction of urban areas, lessening the soil moisture, decreasing the productivity of agriculture and fisheries, and biodiversity disorder^16–18^.

Over the previous 2000 years, the global mean sea level (MSL) did not change by more than a few tens of cm until the start of the industrial era, since then, there have been ample indicators of SLR^20^. This epoch marks the beginning of instrumental tracking of SLR, which has been done by tide gauges (TG) for the past century and by altimeter satellites for the last two decades. The trend of global MSL based on satellite altimetry and TG records is a useful measure of climate change and fluctuations in ocean levels^21^. Besides, the vertical land motion (VLM) could be assessed from the de-seasoned trend of MSL trend (altimetry minus Tide gauges) time series at selected Tide gauges stations^22,23^.

The effects of a 1-meter rise in sea level are anticipated to be most severe along the contemporary Mediterranean coast of Egypt, especially in the vicinity of the Nile Delta^24^. In a limited region of the global ocean, lateral mass transport fluxes control the MSL trend, and Gibraltar mass transfer almost balances the surface water flux in the Mediterranean Sea^21,25^.

Unlike satellite altimeters, which measure sea level changes relative to Earth’s center of mass, TGs record sea level relative to a fixed land point, inherently accounting for land deformations. TG and altimetry data have been employed to estimate VLM along the Mediterranean^3,22,26,27^. However, these efforts faced challenges due to limited TG data availability in the southern Levantine region, especially Egypt’s Mediterranean coast^23^.

^28^, reported that five TG stations, Alexandria, Burullus, Rosetta, Port Said, and Damietta gave a steady mean sea level rise between 1.8 and 4.9 mm/year, with the highest rate at Rosetta and the lowest at Alexandria. Recent altimetric data indicate a 3 mm/year rise in the Mediterranean’s mean sea level from 1993 to 2011^29^. Between 1993 and 2012, this trend averaged 2.44 mm/year^30,31^.

^23^ utilized TG and satellite altimetry to assess SLR in the southern Levantine Mediterranean from 1993 to 2015. Their findings indicated a regional SLR rate of 3.51 ± 0.62 mm/year, exceeding the global mean of 3.19 ± 0.63 mm/year^32^. used geospatial data to analyze recent relative sea level variations, VLM, and absolute SLR along the Nile Delta. Examining TG records and GNSS observations between 1990 and 2016, they found SLR rates of 2.6 to 4.3 mm/year and VLM values ranging from + 0.09 to − 4.3 mm/year.

Thus, the effects of the sea level rise on Egypt’s coastal areas, especially the Nile Delta, are of significant concern for both the Egyptian populace and administration.

Conversely, a continual and relatively slow threat keeps putting pressure on the Nile Delta, where it is experiencing to land subsidence^33–38^. Neotectonics falling, Holocene layers’ compaction, and limited sediment replenishment by greatly decreased Nile flow to Egypt’s shore after constructing of Aswan High Dam in 1968, are processes that contribute to subsidence^39^. Moreover, man-made processes are accelerating the rate of subsidence, including over extraction of ground water^36,40^, construction and urbanization^35,41^, and oil and gas production^36,42^.

Updating the Nile Delta’s deformation maps is urgently needed, as there is still considerable disagreement about the findings of the subsidence rates and patterns along several parts of the Delta plain’s subdivisions and coastal communities through the previous research. Even though numerous studies use the same methods of analysis and time span, there is a definite variability in the findings. The average Holocene subsidence rates in the Nile Delta, which were driven by compacting of Holocene sediments, were measured by^43^ using radiocarbon-dated core samples. The small number of wells analyzed and the absence of temporal and spatial variation in sediment composition, thickness, and texture made it less probable that the predicted subsidence rates would be accurate. The Holocene average subsidence rates solely consider natural variables, but the current rates consider both anthropogenic and natural characteristics^38^^44^. investigated the geomorphological, geologic evolution and subsidence of the Nile Delta, revealing that the rates of long-term subsidence were comparable throughout the Quaternary.

The Nile Delta’s subsidence rates and patterns have drawn significant research interest, primarily using geodetic techniques like GNSS and InSAR. Numerous studies have used GNSS to detect crustal deformation along geodetic networks e.g^34,41,45–48^. , , but findings varied depending on station distribution, temporal coverage, and processing accuracy, showing stable to high deformation rates across cities. Early research, limited by sparse geodetic stations, lacked the spatial coverage of InSAR, which provides broader insights into the Nile Delta’s geodynamics.

InSAR studies include efforts like^49^, , who unsuccessfully used radar interferometry in Cairo due to DEM issues, and^50^, who used PSI with ERS-1 and − 2 images (1993–2000) to estimate a − 7 mm/year deformation rate in Greater Cairo due to tectonics, groundwater extraction, and sediment compaction^38^. calculated − 8 mm/year subsidence at the Damietta Branch, while^37^ found moderate subsidence rates (e.g., El Mahalla: −5 mm/year) using 37 PSI-based ERS-1/2 images^26^. analyzed ENVISAT images (2003–2010) and reported modest subsidence in Alexandria (− 0.4 to − 2 mm/year)^36^. , using PSI stacking of 84 ENVISAT images (2004–2010) and GNSS validation, found subsidence rates ranging from − 0.4 mm/year in the southern Delta to − 9.7 mm/year in the north, with uplift of − 2.5 mm/year in the central Delta^34^. estimated localized subsidence rates of − 6.4 mm/year in Cairo, − 10.3 mm/year in Damietta, and − 4.8 mm/year in El Mahalla using ENVISAT data and GNSS validation^35^. employed Sentinel-1 data (2015–2019) and identified subsidence patterns: urban areas (− 12 to − 20 mm/year), coastal margins (− 3 to − 8 mm/year), and reclaimed lands (− 6 to − 20 mm/year) due to urbanization, sediment compaction, and groundwater overexploitation.

Relying on the above-mentioned results which showed the rates of both SLR and land subsidence of the Nile Delta, the Nile Delta faces significant risks from Mediterranean encroachment due to SLR and land subsidence. Inundation scenarios using various DEMs (e.g., SRTM, ASTER) and Landsat imagery, e.g^8,10,36,39,51–53^. , highlight uncertainties due to DEM limitations. In this study, we employed the high-resolution TerraSAR-X DEM (12.5 m resolution, 3 m horizontal accuracy, and 2–4 m vertical accuracy) from the German Aerospace Centre (DLR) to improve risk assessments.

In this research, we.

- provide a precise assessment of deformation rates and patterns in the Nile Delta by analyzing a series of 191 Sentinel-1 ascending scenes through applying the PSI method, along with validation from eight permanent GNSS stations spanning from 2014 to 2019,
- assess the long-term trends and magnitudes of sea level rise in the Mediterranean and its impact on the Nile Delta employing satellite altimetry data from 1993 to 2020,
- implement the first instance of applying multiple techniques for monitoring land motion at selected sites; measuring the VLM from the trend of de-seasoned sea level variance (altimetry minus TG) time series at the TG site in Damietta City as a case study, and calibrating it with the land motion from InSAR and GNSS at the same TG site,
- generate the Nile Delta GIS-based risk assessment maps (50-100-150 years) by modeling the interplay between land subsidence (from PSI and GNSS) and SLR, and its future socio-economic impacts.

## Geomorphological/geological/structural settings of the nile delta

Geomorphologically, Egypt is the Nile River’s most downstream country that ending with Nile Delta plain. The Nile Delta shoreline stretches approximately 225 km from the west to the east (Alexandria to Port Said). The Nile Delta coastal edge has experienced a northward movement of up to fifty kilometers during the last five thousand years, as documented by^54–56^. The delta plain extends from Cairo in the southern region to the northern Mediterranean coastline, including around 22,000 square kilometers^57^. The delta includes three lagoons, namely Manzala, Burullus, and Idko, which have a connection with the sea^16^. The Suez Canal is located to the east of the Delta and extends into the northeastern coastal area of Lake Manzala. The Nile Delta is a low-lying flat area with varying heights. The southern apex in Cairo is around eighteen meters over sea level, while the northern limit is around one meter over sea level^58^.

**Fig. 2:**
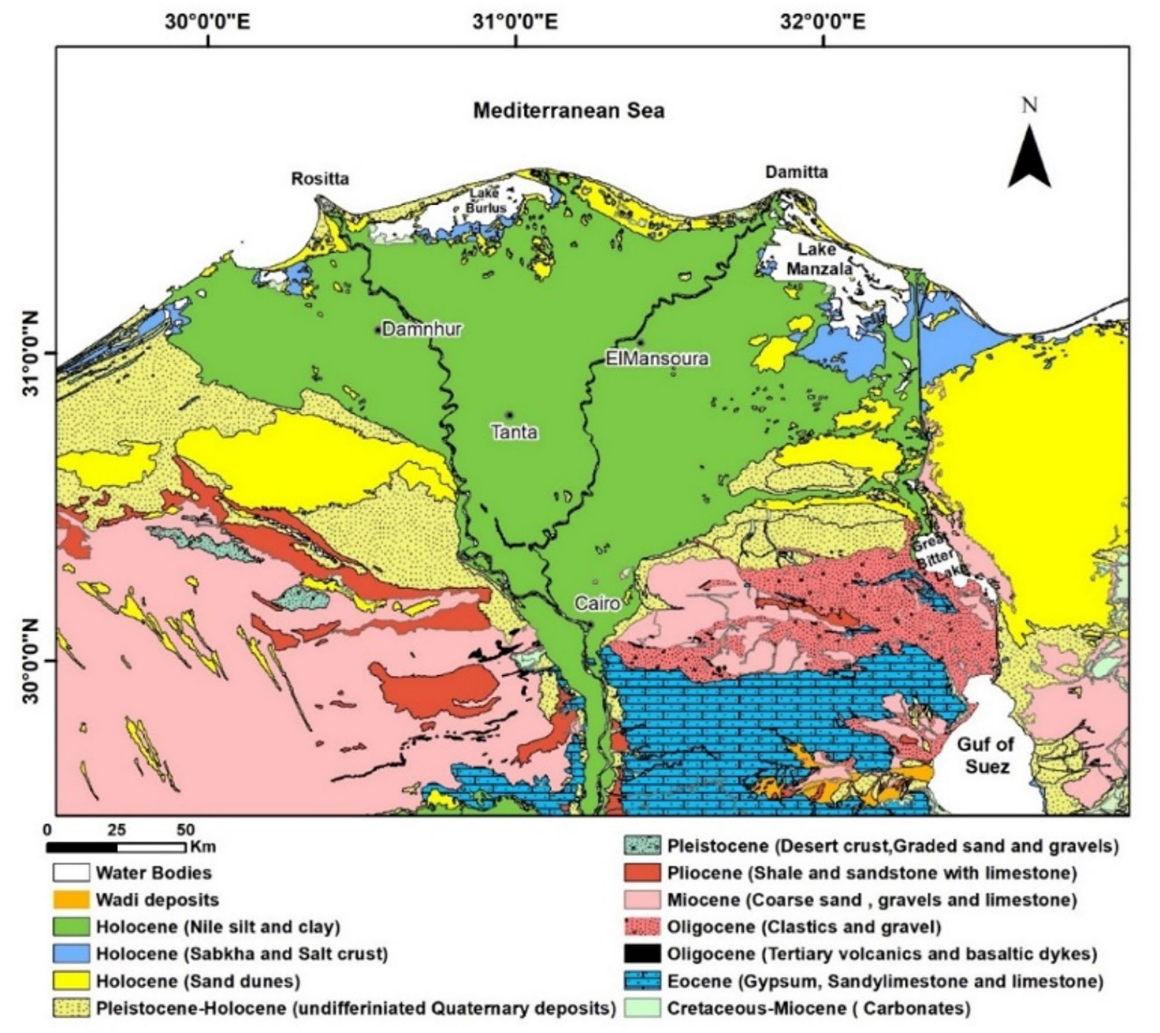
The Nile River Delta’s geological map, modified after^75^. The map was created using ArcGIS, version 10.6.1^76^.

**Fig. 3:**
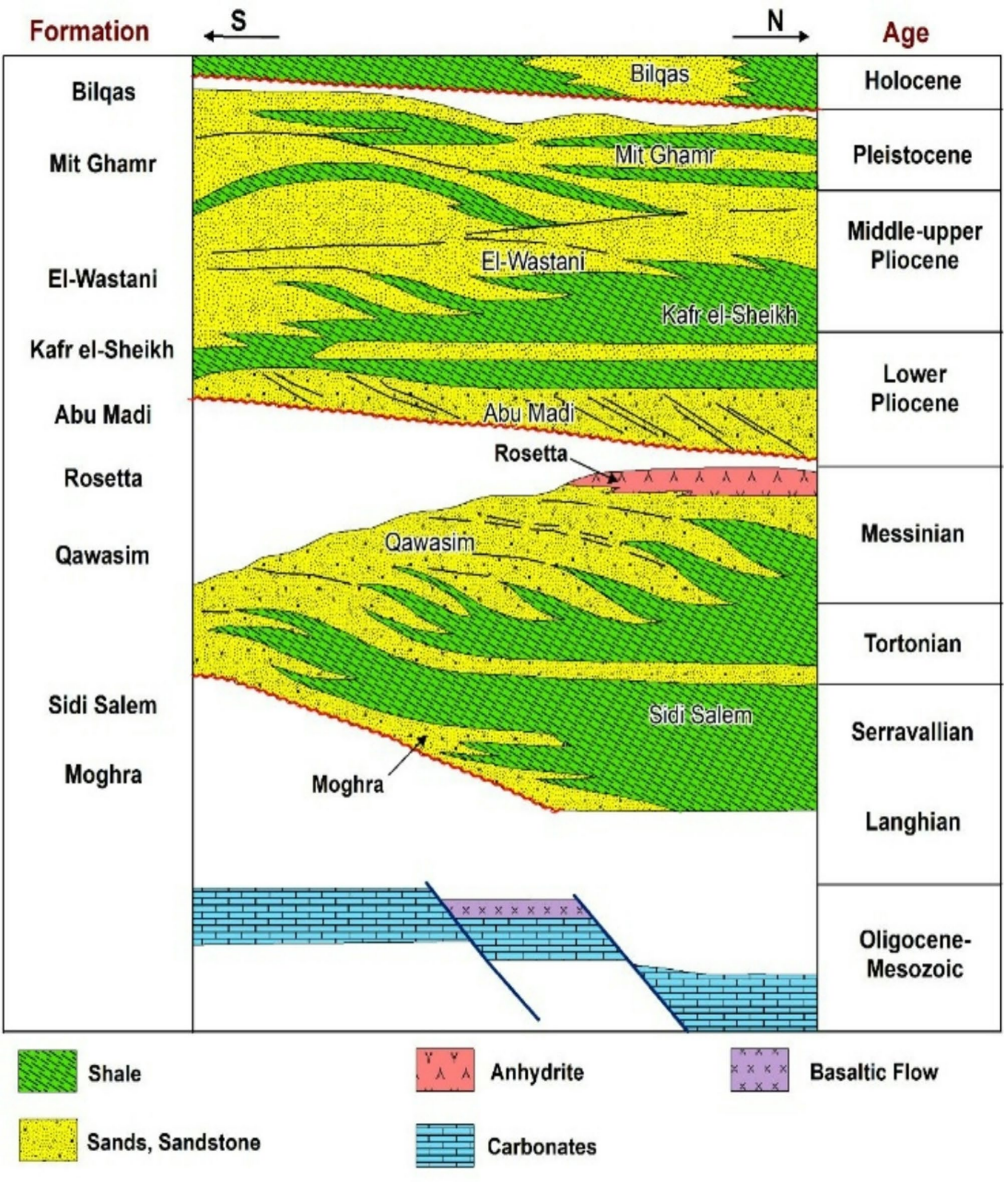
Schematic diagram showing stratigraphic section of the Nile Delta, modified after^44,77,78^, . The figure was created using ArcGIS, version 10.6.1^76^.

Geologically, the Nile Delta in Egypt is considered one of the oldest identified deltaic systems globally^54,59,60^. The Nile Delta, a typical river mouth, was created through the interaction among the Mediterranean Sea coastal erosion and the buildout of River Nile sediments over the course of geologic time^38,61^. It was established through sedimentary processes from Miocene till Holocene (Fig. 2), and constructed by the alluvial deposits driven by the ancient 7 active branches of the Nile [Paleonile (Pliocene), Eonile (Miocene), Prenile (Middle Pleistocene), Protonile (Early Pleistocene), and Neonile (Holocene);^33,58,62,63^. Those distributaries have been posteriorly filled with sediments leaving only the present Rosetta and Damietta branches to become the largest contributors of the Nile Delta sedimentary flux^16,56,64^. The delta’s central hump was formed during the Holocene by a now-extinct branch, Sebennetic, which crossed across the delta’s center, and its surficial geology is dominated by the Neonile that comprising medium- to fine-grained Holocene sand, silt, and clays^16,65–67^, (Fig. 3).

The composition of Nile Delta from thick Quaternary sediments, may lead undergoing natural compaction over time, contributing to land subsidence. The presence of soft clay and silt layers makes it particularly vulnerable to subsidence, especially when influenced by additional factors such as infrastructure development, groundwater extraction, or oil and gas activities. Furthermore, as the land sinks, it lowers the ground level, intensifying the effects of sea level rise. Regions with higher vertical land motion (VLM) rates will experience greater relative sea level rise, heightening the risk of flooding and coastal encroachment.

Structurally, the Nile Delta is situated, at least partially, on a passive continental edges, with its northernmost portion demarcating the transition zone from typical continental crust to oceanic crust of the Herodotus Basin’s^43,68,69^. The hinge zone divides the Nile Delta basin into two structural sub-basins: the southern delta block (SDB), which contains a section of post-Eocene clastics that is between one and one and a half kilometers thick, and the northern delta basin (NDB), which contains a section of Neogene sediments that is between four and six kilometers thick^13,70–72^.

Due to the Nile Delta’s nearly flat topography and its dense population and agricultural use, visually identifying surface expressions of certain faults is impossible. However, the Nile Delta is influenced by regional tectonic activity, including faulting and seismic events that contribute to land deformation. Active faults, such as those growth faults associated with the Mediterranean Ridge and the extensional tectonics of the NDB, may exacerbate localized subsidence^58,73,74^. Seismic activity can also induce ground compaction and trigger further land subsidence, particularly in areas with thick unconsolidated sediments.

## Data and methods

### Assessment of the deformation rates and patterns using PSI method

This study employs InSAR method to measure the long-term subsidence of the Nile Delta and surroundings. Interferometric Synthetic Aperture Radar has recently proven to be more effective, less expensive, independent of atmospheric conditions, and have a wider coverage than conventional ground-based geodetic surveying methods^79–81^. It can accurately and affordably identify the surface ground displacement at millimeter to centimeter precision in a line-of-sight (LOS) vector^82^. Nevertheless, due to spatial and temporal deconvolution and atmospheric delay brought on by various satellite orientations, atmospheric fluctuations, and lengthy observation intervals, the standard InSAR technique is severely restricted^83^. Because of this, PSI^83,84^ was developed to overcome the typical InSAR approach’s incoherence constraint over a wide range of space and time scales.

In this study, we utilized Sentinel-1 A and 1B satellites using the interferometric wide-swath (IWS) imaging mode and level 1 C-band (SLC) data format.

- **Ascending track T58-A** was selected, covering **frames 93 and 98 (**Fig. 1**)**.
- The satellites’ **revisit times vary between six and twelve days**, with some regions requiring less time than others.
- The retrieved scenes (**a stack of 191 scenes**) were acquired from **2014 to 2019**.
- The data was obtained from the **Alaskan Satellite Facility (ASF) Distributed Active Archive Center (DAAC) (accessed in August 2021)**.

**PSI Processing Workflow**.

- PSI processing of the Sentinel-1 scenes was performed using the **commercial SARscape software 5.5** (SARscape), an **ENVI plug-in written in C++**. SARscape was selected for PSI processing due to its robust capabilities in handling large-scale interferometric datasets and its seamless integration with ENVI, allowing for efficient processing and visualization. Additionally, SARscape offers advanced phase unwrapping algorithms, precise atmospheric corrections, and optimized persistent scatterer selection, making it well-suited for analyzing long-term ground deformation with high accuracy. Furthermore, it is one of the most commonly used and well-known software tools for InSAR processing, ensuring reliability and familiarity within the geospatial research community.

**Preprocessing**.

- The **IW-SLC scenes of the two frames** were:
  - **Imported to SARscape**.
  - **Mosaicked**.
  - **Orbital correction applied**.

**Interferometric Stacking**.

**Fig. 4:**
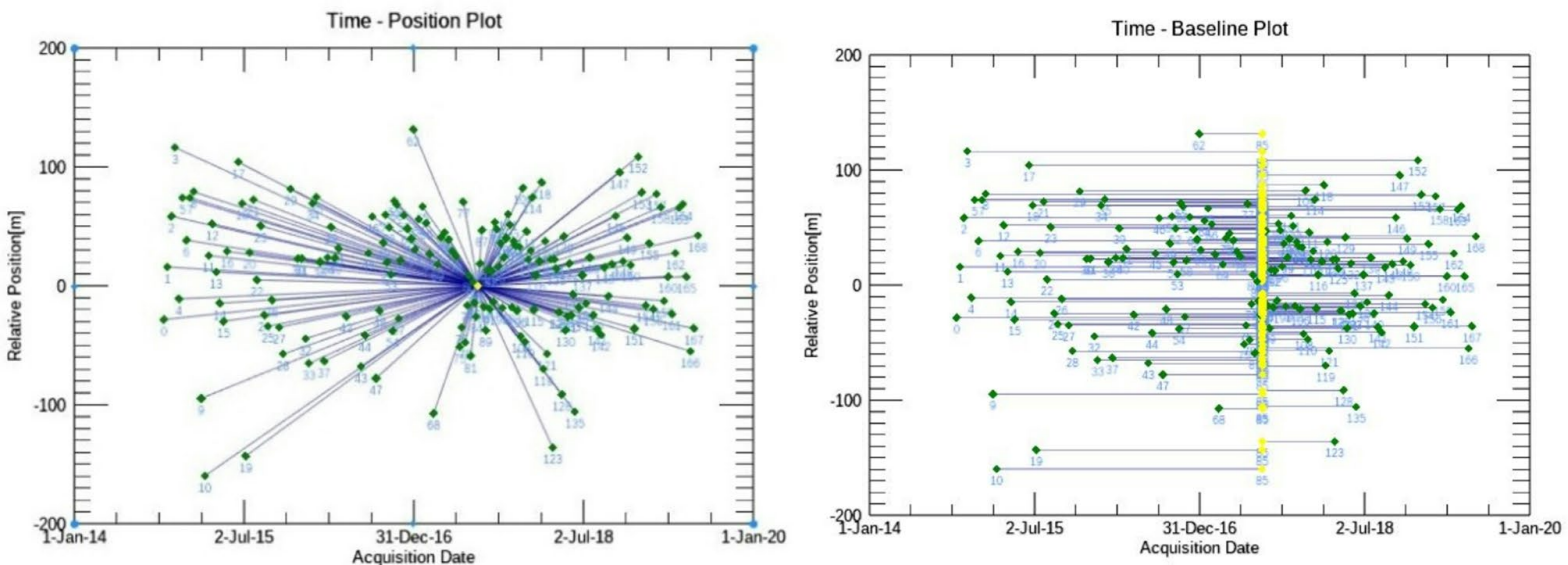
Spatial base-line time plot for the ascending track (190 scenes) (PSI-connection graph), the PSI-master scene is plotted in yellow colour. The figure was created using SARscpae software, version 5.5., plugged in ENVI, version 6.1^85^.

- Several phases of **interferometric stacking** were initiated **after orbital correction**.
- The **creation of the connection graph (**Fig. 4**)** was performed, which describes:
  - The **SAR pair combination (Master and Slaves)**.
  - The **connection network** designed to obtain **multiple differential interferograms**.
- **Master and Slave selection constraints**:
  - **Spatial baselines** between the master and slave scenes must be **less than 1**,**300 m**.
  - **Temporal baselines** must be **as near to zero as feasible**.
  - **Doppler centroid mean frequency differences** between the master and slave must be **as close to zero as possible**^83^ .

**Interferometric processing**.

- The **PSI interferometric process** allowed the following steps:
  - **Co-registration** of all slave scenes to the master reference scene.
  - **Interferogram creation and flattening**.
- **Co-registration**:
  - Compensates for **scale discrepancies between the master and slave scenes**.
  - Ensures **proper pixel superimposition in slant-range geometry**.
- **Interferograms** were generated among the master and slave scenes.
- **Flattening**:
  - Flattened to the **DEM (TerraSAR-X**,** 12.5 m resolution**,** 2 m accuracy)** from the **German Aerospace Centre (DLR) TerraSAR-X/TanDEM-X Satellite**.
  - Projected in **slant-range geometry onto the master scene**.
  - Output: A **flattened interferogram devoid of constant and topographic phase**.

**Displacement velocity estimation**.

- The **first inversion model** was implemented to:
  - **Preliminarily calculate the residual displacement velocity and height**.
  - Identify **coherent radar signal reflectors (Persistent Scatterers-PS)** such as:
    - **Metallic and concrete features**.
    - **Bridges**,** dams**,** roofs**,** poles**.
    - **Well-exposed outcropping rock formations**.
- The **second (final) inversion** was run on the first linear model products to:
  - Derive **date-by-date displacements**.
  - Subtract the derived displacements from **interferogram measurements**.
- The **atmospheric phase** was eliminated from the **subtracted component** by:
  - **Applying a low-pass filter** to analyze the **spatial distribution of atmospheric variations**.
  - **Applying a high-pass filter** to analyze the **temporal distribution**.

**Final Steps & Geocoding**.

- **Geocoding** was applied to the **PSI products** with a **coherence threshold of 0.3**. Since the Nile Delta is a heavy vegetated area, the coherence threshold should not be lower than 0.3.
- The resulting **displacement values** are in the **LOS direction**, because only **ascending Sentinel-1 scenes** were processed.
- **Horizontal motion in the Nile Delta is negligible**.

### Extraction of the vertical velocity across geodetic stations

The NRIAG established the Egyptian Permanent GNSS Network (EPGN) which covers Egypt with special focus on active regions such as the Nile delta. Eight EPGN stations are situated in the Nile delta, demonstrated in Fig. 1. In this work, we used the data collected from these eight stations for the period 2014–2019. It is worth mentioning that even the density of the GPS station is low, it provides a vaulabe source for validating the PSI results from InSAR time series analysis. Due to the processing power limitations, we have processed three months per year (January, July, and December). The processing of the GPS dataset collected between 2014 and 2019 was performed by Bernese V.5.2 software^86^, with the satellite clocks, IGS final orbits, Earth orientation parameters, and The IGS14 antenna phase correction. Aside from the EPGN stations, we incorporated Fifteen permanent stations from the International GNSS Services (IGS), out of which Thirteen are part of the ITRF2014 and were utilized as reference points for defining the datum. The data obtained from EPGN stations in the Nile delta were analyzed using the IGS final orbits, Earth orientation parameters, satellite clocks, and the IGS14 antenna phase correction.

We implemented the processing approach outlined by^87–90^.

The processing was conducted on a daily basis using:

- Bernese 5.2 (Dach et al. 2015) for GNSS observations processing,
- the Ionosphere free linear combination L3,
- Dry Niell as the troposphere model,
- an elevation cut-off angle of 3°,
- International GNSS Service (IGS) absolute phase centre corrections,
- IGS final orbit products,
- The Bernese combination tool (ADDNEQ2) was used to estimate the velocity and position of all processed stations,
- To obtain a clean solution, we applied an outlier detection/rejection procedure. Subsequently, the refined solution was utilized to estimate the updated set of station positions and velocities. For better estimates of the standard deviations, we estimated scaled standard deviations by the computing a ratio of the formal standard deviation (resulted from Bernese) and repeatability assessed by the combination step^87–89^. Moreover, we have used the time series available by SONEL (Société Nationale de Localisation des Réseaux) for the GPS station, which is located in Alexandria, and.
- 13 stations used for the datum definition, ITRF2014 datum^91^.

### Projection of sea-level changes using satellite altimetry data

**Fig. 5:**
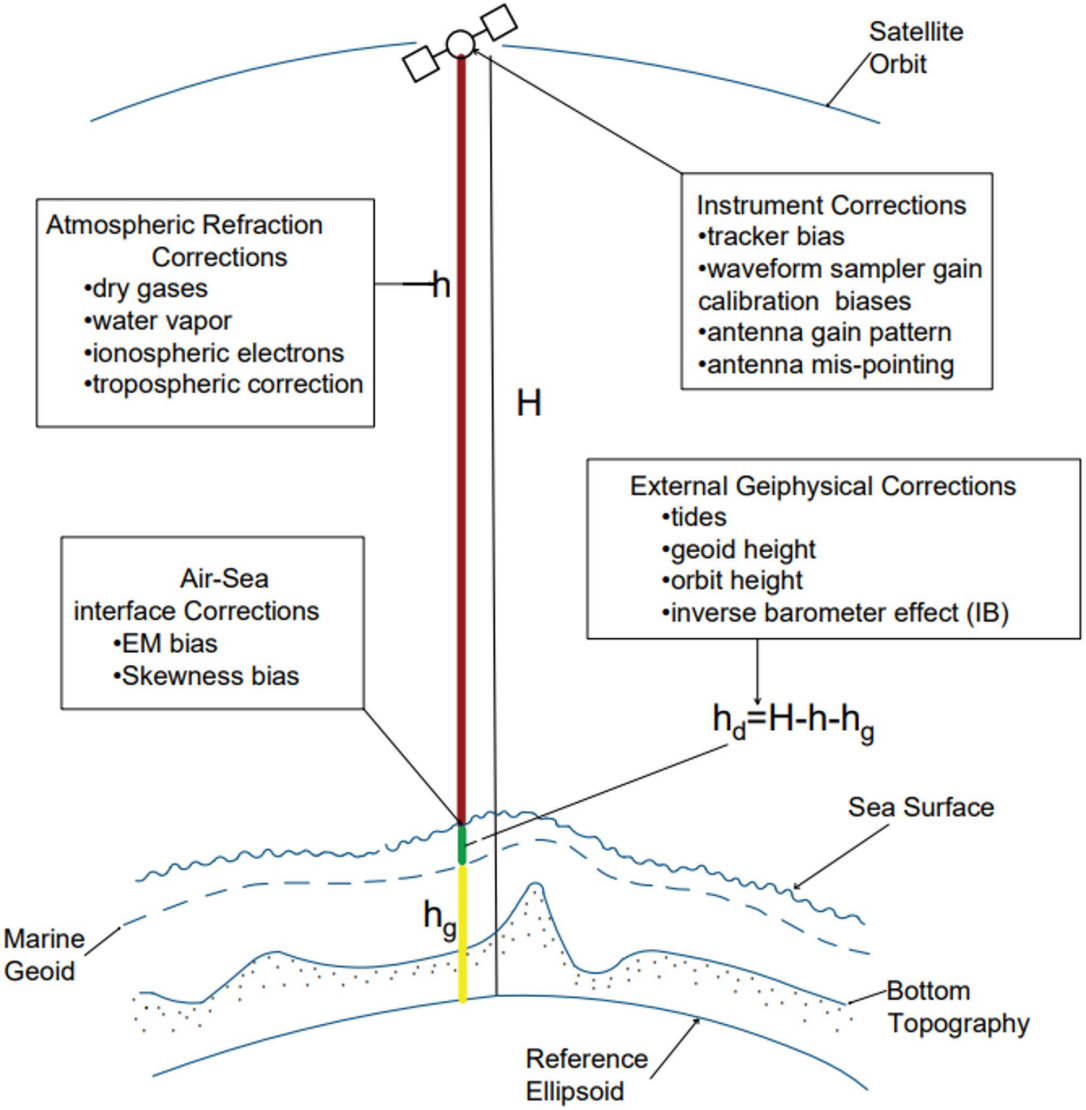
A visual depiction illustrating the measurements from an altimeter and the necessary corrections to obtain the dynamic sea surface increase, denoted as h_d_. The altimeter range is represented ash, while H and hg correspond to the orbit geoid height and height, respectively. These values are referenced relative to an ellipsoid approximation of the Earth’s surface, as adapted from the work of^96^.

Satellite altimetry measures the round-trip duration of a radar pulse from the satellite to the target surface in order to compute the distance between them. The fundamental altimetric measurement is the average dynamic topography of the ocean’s surface, which is used to calculate the sea surface heights and the geostrophic surface currents. Altimetry is a method used to determine the elevation of a surface in relation to a geodetic reference ellipsoid, originally described by^92^. This study employed the gridded sea level anomaly data from the Copernicus Marine Environment Monitoring Service (CMEMS) to cover the Nile Delta region in the Southern Eastern Mediterranean (data accessed in June 2023). The research area spans from 31° S to 33° N latitude and 28° W to 33.25° E longitude. The dataset is accessible at a geographic resolution of 1/8-degree and encompasses the time span from January 1993 to December 2020. The data set has undergone multiple corrections (Fig. 5), which include: (a) addressing instrumental errors such as tracker bias, antenna gain pattern, waveform sampler gain calibration biases, and antenna mis-pointing; (b) correcting for biases in the electromagnetic (EM) and skewness at the air-sea interface; (c) adjusting for atmospheric effects such as ionosphere, troposphere, dry gases, and water vapor; and (d) compensating for geophysical effects like tides, geoid height, orbit height, and the inverse barometer effect (IB). The altimetric measurements were adjusted for the significant impact of the Inverse Barometer (IB) correction using the dynamic atmospheric correction (DAC) method, as described by^93^. The DAC takes into account both the atmospheric pressure and wind influences, as described by^94^. The altimetric data were transformed into monthly averages in order to maintain consistency with the TG data. We eliminated the seasonal variation from altimetry data by employing a harmonic approach. Next, the linear trend of sea level anomaly (SLA) was calculated for the time series after removing the seasonal variation using the usual least square method^95^.

### Evaluation of the VLM using the combination of altimetry and TG

Tide gauges are a widely used method for continuously measuring sea level in coastal areas. They are typically located on piers in harbors and serve historical and navigational purposes by measuring sea level relative to a nearby geodetic reference point (i.e., benchmark). The global tidal gauge network is managed by the National Oceanography Center’s Permanent Service for Mean Sea Level (PSMSL)^97^, and a free download of the global TG sea level information is available. Nevertheless, the tidal gauges that are accessible in Egypt, especially, and around the coast of North Africa are few and quite hard to reach. Like satellite altimetry, TG data are affected by some errors that have to be taken into consideration and fixed; such as geophysical errors that might be caused by atmospheric loading and VLM. While the atmospheric error is resolved through the IB correction with sea level pressure provided by Boulder, NOAA/OAR/ESRL PSD, USA, Colorado, via their website at http://www.esrl.noaa.gov/psd/, the vertical motion is resolved using continuous GPS or gravity measurements.

In addition, the effect of glacial isostatic adjustment (GIA) was removed from both altimetry and tide gauges as described in^90,98,99^. The GIA correction represents the slow part of the Earth’s response to the redistribution of mass after the last deglaciation and the changes in the shape of the ocean basins. Here, we used the rate of relative sea level rise (dSea) and the rate of change of geoid height (dGeoid) from the ICE-6G-C (VM5a) model^100^ to correct the tide gauge and altimetry data, respectively.

**Fig. 6:**
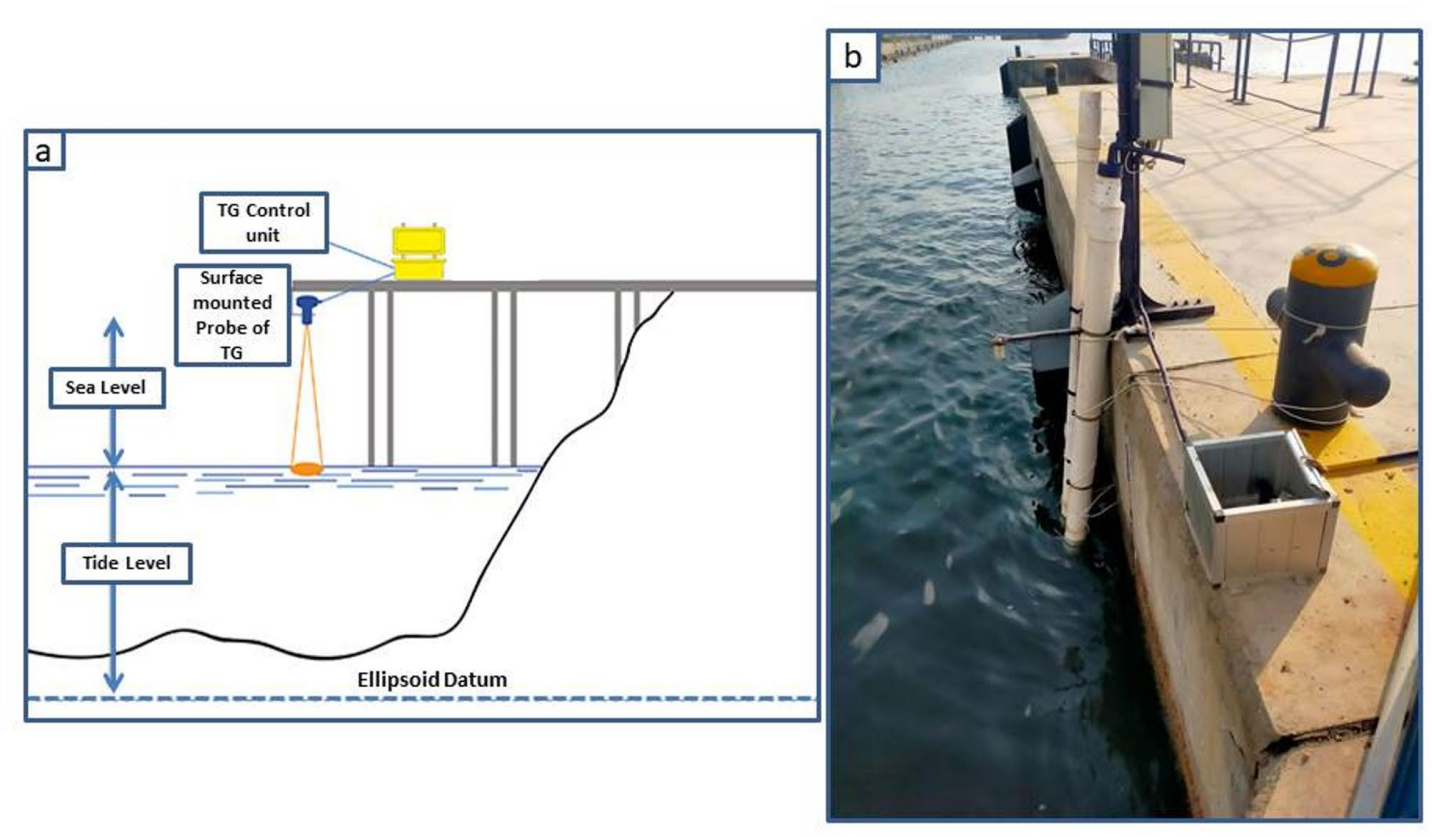
(**a**) Scematic diagram of TG system, modifed after^101^, ; (**b**) the TG unit in Damietta, Egypt.

In this study, the monthly MSL anomalies data from only 1 TG station was used from New-Damietta city during the period (1997–2019). The sea level data is derived from the hourly record collected from the Hughes mechanical TG (Fig. 6) located in Damietta. This data is provided by the Coastal Research Institute in Egypt.

To ensure data quality, the TG data sets underwent a quality control process involving the analysis of time series in one-month segments. Data values that exhibited a variation exceeding two Standard deviations from the mean were detected and then eliminated from the time series. In instances where there were gaps in the data spanning a few months (less than two months), a linear interpolation method was employed to replace the missing values.

The altimetry datasets were initially adjusted for the Digital to Analog Converter, as mentioned earlier. In order to align the TG data with the altimetry data, the DAC was removed^23,102^. The Auxiliary items dataset, available for free on the Archiving, Validation, and Interpretation of Satellite Oceanographic (AVISO) website, was utilized to retrieve the DAC data. The data was collected at tide gauge stations with a spatial grid resolution of 0.25°*0.25° and a time resolution of 6 h. The dataset can be accessed from the AVISO website (http://ftp.aviso.altimetry.fr). The data were aggregated on a monthly basis to maintain the same temporal resolution as the TG data. A time series of altimeter data has been selected for the Damietta TG station, which corresponds to the nearest point in the multi-mission altimeter grid. In order to generate monthly anomalies without seasonal variations, the average seasonal patterns for both the altimeter and TG time series were calculated separately for each grid point. These seasonal patterns were then subtracted from the monthly average of each dataset. In order to avoid the inclusion of biases, the average seasonal pattern was determined by estimating the average monthly value for each month, using only complete years within the specified analysis period. In order to obtain monthly values without seasonal variations, the average value of each month was removed from all comparable months over all years. The linear trend of the residuals has been independently computed at each grid point utilizing the usual least squares method for both the altimeter and tide gauge data.

The sea level measured by a TG station is influenced by the Earth’s crust and is therefore affected by VLM. On the other hand, the sea level measured by altimetry is relative to the geo-center and is not influenced by VLM. The VLM contributes to the overall long-term discrepancy in sea level between TG and satellite altimetry^23,103–105^. Therefore, the VLM rates have been calculated from the trend of de-seasoned sea level difference (altimetry minus TG) over time at the Damietta TG sites^103,105^.

### Estimate the anticipated inundation scenarios

**Fig. 7:**
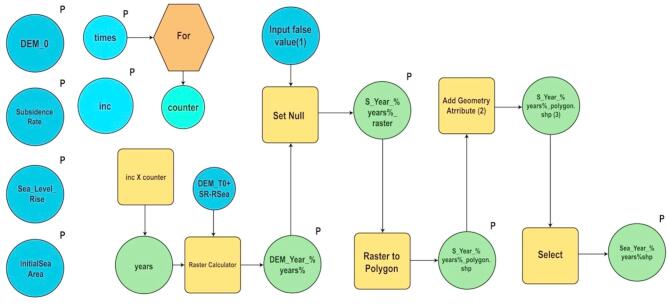
The model workflow used to automate sea encroachment mapping based on altimetry, PSI interferometric results, and TanDEM-X.

Global MSL rise will drive impacts and adaptation demands along the world’s coasts over the 21st century and beyond. Creating a scenario of local relative SLR to assist in impact assessment and adaptation planning is a crucial step in evaluating these challenges. The problem is Egypt’s Nile Delta is subsiding versus the Mediterranean SLR. Because of the low land that is located in the northern coastal zone of the Nile Delta, it is susceptible to both indirect and direct effects of sea level rise brought on by climatic changes, especially inundation. According to the cautious approach, evaluation and analysis of the implications are still required regardless of the uncertainty surrounding generated scenarios for climate alteration and expected sea level rise. The findings of PSI interferometry were incorporated into our Geographic Information System-based risk evaluation for the coastal zone of the Nile Delta under a variety of SLR scenarios, SLA trend from altimetry and TanDEM-X {resolution: 12 m, relative horizontal accuracy: 3 m, and relative vertical accuracy: two meter (slope ≤ 20%), four meter (slope ≥ 20%)}. We have used the Arc-GIS model (Fig. 7) to automate sea encroachment mapping. ArcGIS models consist of workflows that connect sequences of geoprocessing tools, with the output of one tool serving as the input for another.

For data preparation, it was necessary to be sure that the elevation at shoreline was close to zero, and elevation over sea was zero. Uplift in the deformation product was disregarded, and any positive value was simply assigned to zero (stable). To interpolate point subsidence data to raster data, we employed the spatial analyst interpolation method known as Inverse Distance Weighting (IDW). Soil types were not explicitly considered in the inundation model. The focus was on integrating elevation changes (subsidence) and sea level rise to estimate potential flooding areas. However, future work could incorporate soil properties to refine flood susceptibility assessments.

**Workflow components (**Fig. 7**)**:

1. Inputs (Blue Circles, “P” labeled):

- DEM_0 (Initial Digital Elevation Model).
- Subsidence Rate.
- Sea Level Rise.
- Initial Sea Area.

2. Processing Steps:

- For Loop (Orange Hexagon): Iterates through time steps.
- Counter (Cyan Circle): Keeps track of iterations.
- Raster Calculator (Yellow Rectangle): Computes new DEM values using the equation “DEM_T0 + SR - RSea”.
- Set Null (Yellow Rectangle): Removes false values.
- Raster to Polygon (Yellow Rectangle): Converts raster results to vector polygons.
- Add Geometry Attribute (Yellow Rectangle): Adds geometric attributes to the polygons.
- Select (Yellow Rectangle): Extracts sea-encroached areas for further analysis.

3. Integration of IDW Interpolation:
4. To refine the spatial analysis, we employed the Inverse Distance Weighting (IDW) interpolation method from the Spatial Analyst toolset. IDW is a widely used geospatial interpolation technique that estimates unknown values based on the values of nearby known points. This method assumes that points closer to each other have more similar values than points farther apart.IDW is a deterministic interpolation technique that predicts values at unknown locations using a weighted average of nearby data points. The weight assigned to each known point is inversely proportional to its distance from the unknown point.
5. Final Outputs (Green Circles, “P” labeled):

- S_Year_% years%_raster (Intermediate raster result).
- S_Year_% years%_polygon.shp (Polygon shapefile representing sea encroachment for a given year).
- Sea_Year_% years%shp (Final selected encroachment shapefile).

## Results and discussions

### Deformation of the nile delta by PSI

**Fig. 8:**
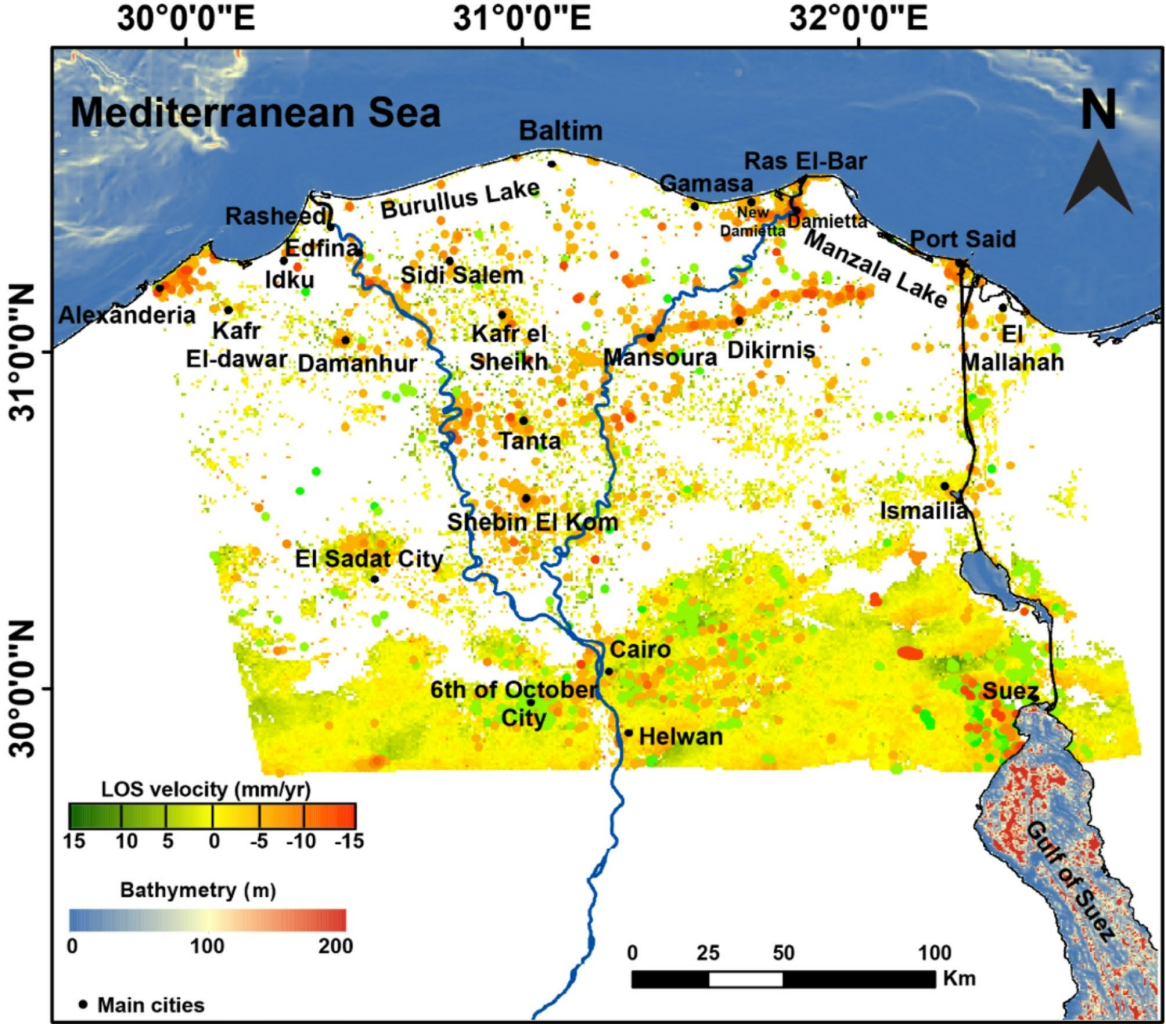
Deformation rate across the Nile Delta of Egypt and its surrounds using the PSI technique over the period (2014–2019), from Sentinel-1 data analysis. The map was created using ArcGIS, version 10.6.1^76^.

PSI technique had been used to quantify the subsidence rates throughout the Nile Delta through the time span (2014–2019) (Fig. 8). The delta has a dense vegetation, which causes random surface and volumetric backscattering, which leads to significant incoherence. Therefore, the deformation estimation of the Nile Delta was more evident and apparent within urban areas where PSI works well, where the consistent coherence due to the ability of detecting multiple PS points per pixel. Since the processed track was just the ascending one, the generated velocities were expressed as LOS (Fig. 8).

**Fig. 9:**
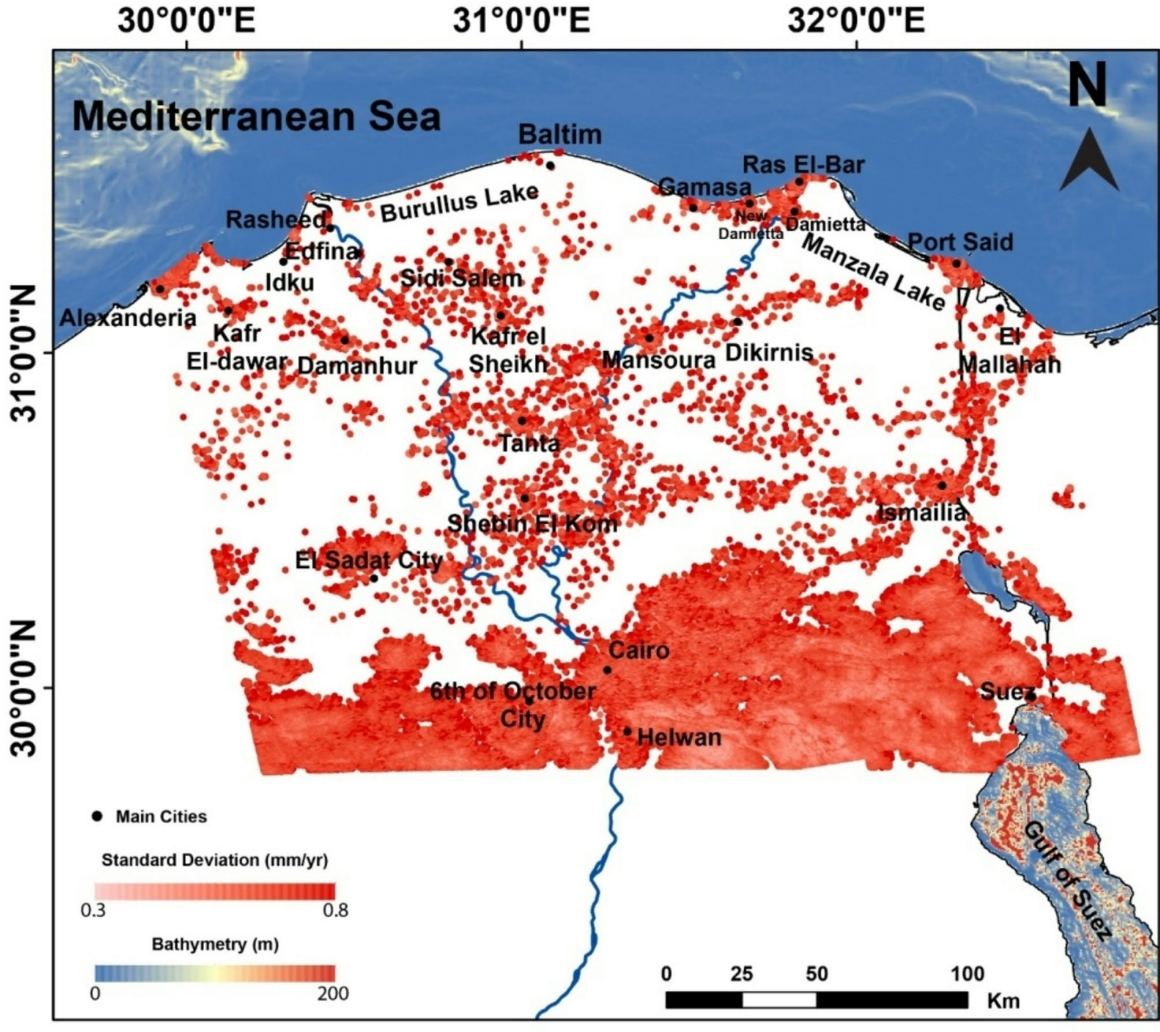
The standard deviation of PSI results, varying from 0.3 to 0.8 mm/year, the hot red coulor refering to the higly standard deviation, while the pale red indicating the higher certanity regions. The map was created using ArcGIS, version 10.6.1^76^.

The PSI findings showed that the general pattern of the Nile Delta is nearly stable, and scattered moderate to high values of subsidence are located in major cities. The distribution of high velocities (subsidence rtaes) was mostly dispersed within the big cities in the Nile Delta and along the Nile 2 active branches cities or in the direction of their peripheries (Fig. 8). There is also obvious moderate subsidence from Mansoura to Dikirnis till Manzala Lake and then Port Said. In addition, the area between Cairo-Suez, the western side of the Delta, is showing alternative uplifts and subsidence, which could be evident to the complicated pattern of structure in this area, which needs more and detailed study. The standard deviation of the PSI pixels was calculated (Fig. 9), and showed a reasonable value (0.3–0.8 mm per year) indicating great accuracy of the analysis and results. The highest accuracy (lowest standard deviation values ~ 0.3–0.4 mm per year) was located in the desertic areas, southeast of the Delta, where as the higher rates of standard deviation (~ 0.6–0.8 mm/year), this is due to the higher coherence in desertic areas than the vegitated ones (Fig. 9).

### Deformation of the nile Delta by GNSS

**Fig. 10:**
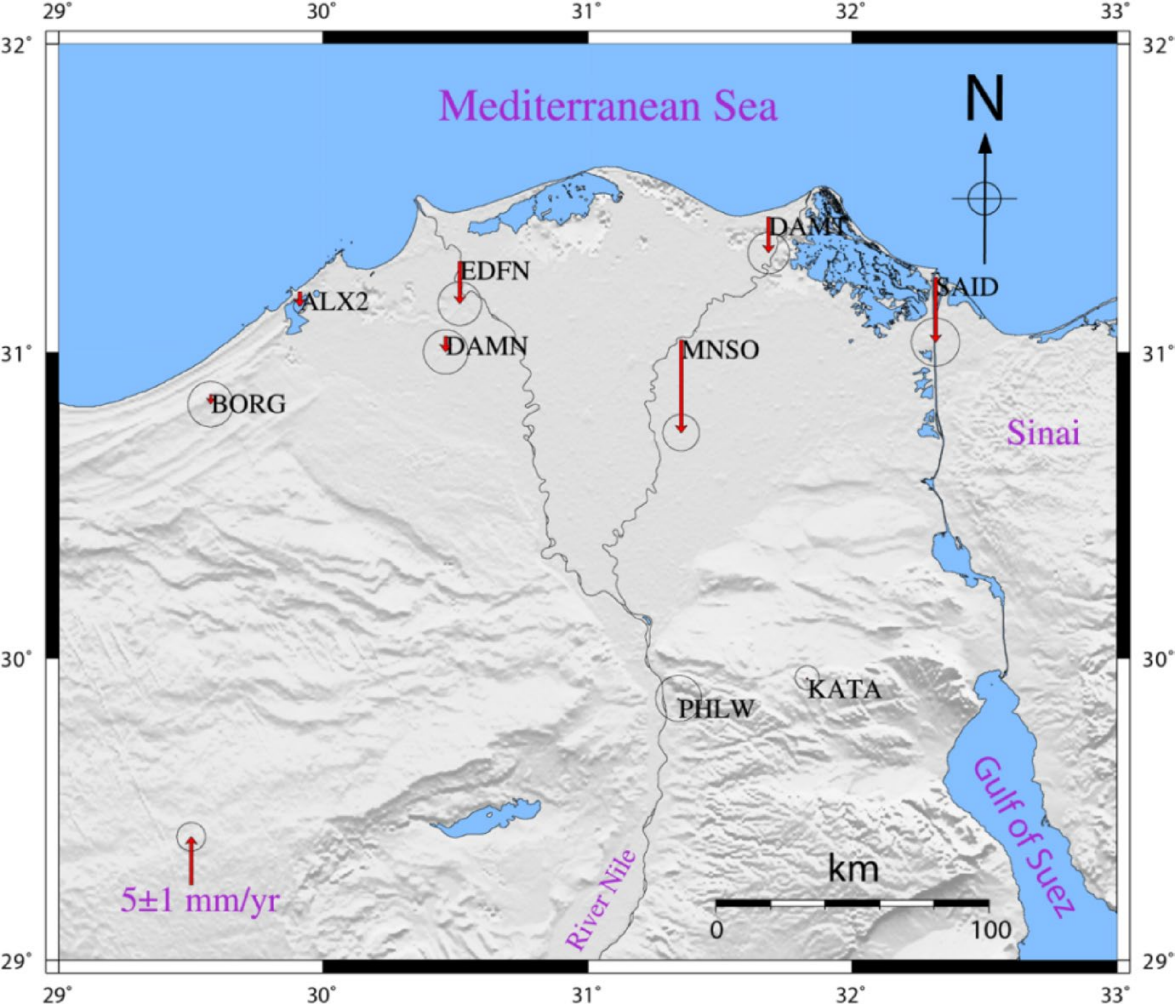
Vertical velocity field for the Global Positioning System stations within the Nile delta. Thae map was created using Generic Mapping Tools (GMT), version 5.

As exhibited in Fig. 10; Table 1, The GPS results of the Up component showed that almost all the GPS stations in the northern part of the Nile delta have a general trend of subsidence. The rate of subsidence is different from one station to another. The results of the Global Positioning System stations illustrate that all GPS sites are suffering from a general trend of subsidence with various rates. Stations (Alexandria-ALX2, Damanhor-DAMN, Edfina-EDFN, Mansoura-MNSO, New-Damietta-DAMT, and Port-Said-SAID) show significant rate of subsidence of -1.5 ± 0.4 mm per year, -1.6 ± 1.5 mm per year, -4.4 ± 1.5 mm per year, -9.6 ± 1.3 mm per year, -3.1 ± 1.4 mm per year, and − 6.7 ± 1.7 mm per year, correspondingly. The rest of the GPS stations (BORG, PHLW, and KATA) do not show significant velocity rates in the up component with 95% of the confidence level (BORG − 1.0 ± 1.6 mm per year, PHLW 0.2 ± 1.6 mm per year, KATA 0.2 ± 0.8 mm/year).

**Table 1:** The location of GPS sites processed in this work, in longitude and latitude and velocities in ITRF2014 for the up component with scaled standard deviations. (*) is for stations located in the nile delta.

Station	Longitude	Latitude	Vu (mm/year)	σu (mm/year)
ADIS	38.77	9.04	-2.30	2.28
ANKR	32.76	39.89	-3.89	1.46
BHR3	50.61	26.21	0.78	1.32
BHR4	50.61	26.21	0.68	1.15
BORG*	29.57	30.86	-1.02	1.58
BSHM	35.02	32.78	1.19	1.58
DAMN*	30.46	31.05	-1.64	1.55
DAMT*	31.68	31.44	-3.73	1.45
EDFN*	30.52	31.30	-4.39	1.52
GRAZ	15.49	47.07	1.34	1.50
IISC	77.57	13.02	-1.74	1.94
ISBA	44.44	33.34	0.94	1.74
KATA*	31.83	29.93	0.22	0.84
MAL2	40.19	-3.00	-0.47	2.23
MARS	5.35	43.28	-1.33	1.61
MAS1	-15.63	27.76	-1.18	1.49
MBAR	30.74	-0.60	0.99	1.89
MNSO*	31.35	31.04	-9.65	1.27
PHLW*	31.34	29.86	0.16	1.60
RAMO	34.76	30.60	1.80	1.30
SAID*	32.31	31.25	-6.77	1.68
SOFI	23.39	42.56	-0.19	1.62
TEHN	51.33	35.70	-3.06	2.24
YEBE	-3.09	40.52	0.73	1.17
ALX2*	29.91	31.20	-1.50	0.45

### Deformation of the Nile Delta by PSI and colocated GNSS

**Fig. 11:**
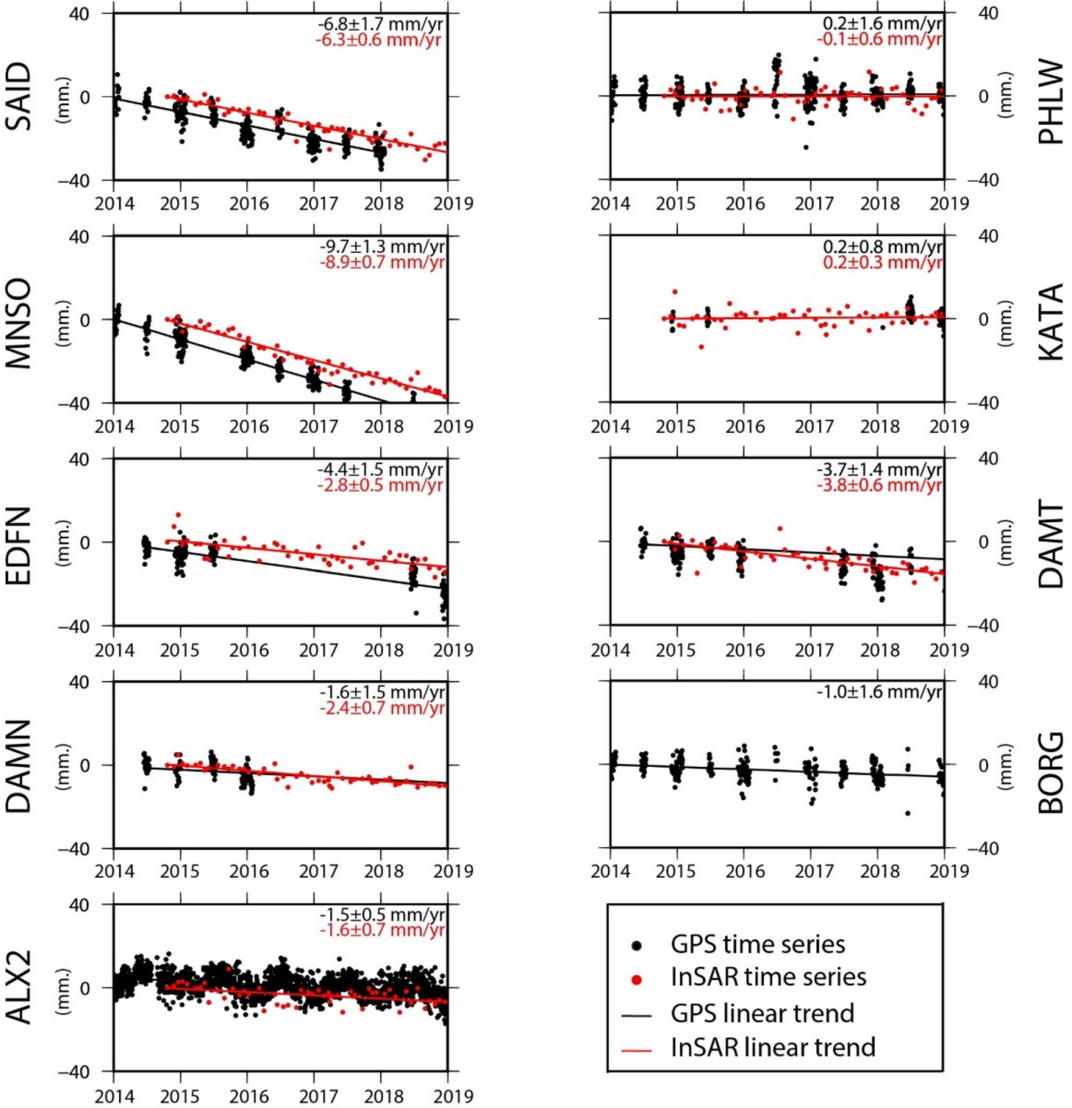
Time series GPS sites and PSI pixels co-located with the GPS stations in the Nile delta, and showing good correlation pattern. This figure was created using Generic Mapping Tools (GMT), version 5^19^.

The denesity of PS pixels is very high for the southern part of the study area, as it is mainly desert and hard rocks. The denesity of pixel is moderate for the cities within the Delta, while it is very low for the vegetated areas in the Nile Delta, as presented in Fig. 8. For security reasons, the GNSS stations are located in the main cities within the Delta. In comparison to the PS pixels, the denesity of the GNSS station is low but there are PS pixels which are co-located with the GNSS station and used for the comparison of the estimated rates from PSI and GNSS. The results of the deformation rates obtained from the PSI pixels closing to the GNSS sattions were apparantely compared revealing consistency between the satellite technique (InSAR) and the ground-based one (GNSS), and it could be summarized as follow (Fig. 11); (1) the subsidence rates were − 6.8 ± 1.7 mm/year from GNSS and − 6.3 ± 0.6 mm/year from PSI in Port Siad city (SAID); (2) the subsidence rates were − 9.7 ± 1.3 mm/year from GNSS analysis and − 8.9 ± 0.7 mm/year from PSI in Mansoura city (MNSO); (3) the subsidence rates were Edfena (EDFN) were − 4.4 ± 1.5 mm/year from GNSS and − 2.8 ± 0.5 mm/year from PSI; (4) the subsidence rates were − 1.6 ± 1.5 mm/year from GNSS and − 2.4 ± 0.7 mm per year from PSI in Damanhor city (DAMN); (5) the subsidence rates were − 1.5 ± 0.5 mm per year from GNSS and − 1.6 ± 0.7 mm per year from PSI in Alexandria city (ALX2); (6) almost stable deformation rates in Helwan city (PHLW), 0.2 ± 1.6 mm/year from GNSS and − 0.1 ± 0.6 mm per year from PSI; (7) the same value from GNSS and PSI in Kattamia city (KATA) 0.2 mm/year, with different standard deviations, ± 0.8 in GNSS and ± 0.3 for PSI; (8) the subsidence rates were − 3.7 ± 1.4 mm per year from GNSS and − 3.8 ± 0.6 mm/year from PSI in New-Damietta city (DAMT); and (9) the GNSS velocity for Borg Elarab city (BORG) was − 1 ± 1.6 mm/year. The maximum variance among the results of the two techniques was around − 1.6 mm/year in EDFN which could be regarded to a local effect at the GNSS station. On the other hand, the results of KATA, DAMT, PHWL, ALX2 were almost similar with insignificant differences. Such a result shows the consistency of the achieved results from both PSI analysis and GNSS rates.

Such disoriented subsidence seems to be mostly caused by human activities, for example urbanization growth, the production of oil and gas, and underground water extraction, rather than being structurally regulated.

According to^36^, PSI stacking of 84 ENVISAT SAR images from 2004 to 2010 over the entire delta revealed a consistent and mild subsidence with a magnitude of − 0.4 mm per year in the southern part of the Delta. They also observed a regional uplift of − 2.5 mm per year in the delta’s plains linking to the hinge zone, and the delta’s northern section experienced a notable subsidence rate that may reach − 9.7 mm per year. Moreover^41^, based on studying a number of GPS stations in the Nile Delta between 2013 and 2017, they concluded that the northern part of the Nile Delta suffers from clear subsidence ranging from − 2 to − 8.5 mm per year, while the central part of Nile Delta suffers from clear uplift as represented by Tanta (11 mm/year), Damnhour (27.9 mm per year) and Hamoul (1.9 mm per year) station. In addition^69^, from studying the subsuface structure of the Nile Delta using satellite gravity data (GOCO-06) which was constrained by seismic data. They disclosed that a twenty- to forty-kilometer-wide flexural zone with an east-west trending hinge zone divides the NDB from the SDB, separating the two regions of the Nile Delta. This hinge zone is characterized by a rapid and abrupt thickening in sediments towards north (4–6 km north and almost 1 km south). Theoretically, this load of sediments in northern delta basin could result in a significant amount of subsidence towards North and a degree of stability towards South. In our study, we did not relate or correlate such a surficial regional deformation pattern that connected to the deep-seated structures of the Nile Delta^69^, nor the pattern derived from the study of^36,41^. And the obtained pattern from our PSI result (Fig. 8) is connected to major cities and villages, in accordance with the findings of^34,35^. In contrary^48^, , based on studying some GPS stations from 2013 to 2020, he reported considerable subsidence rates along the Delta northern part (average − 4.53 mm/year) and the highest subsidience rates in the central part of the Nile Delta (average value − 9.10 mm/year). These discrepancies in the overall deformation pattern of the Nile Delta are challenging, necessitating an increasing amount of research to attempt and determine the causes of these variations.

**Fig. 12:**
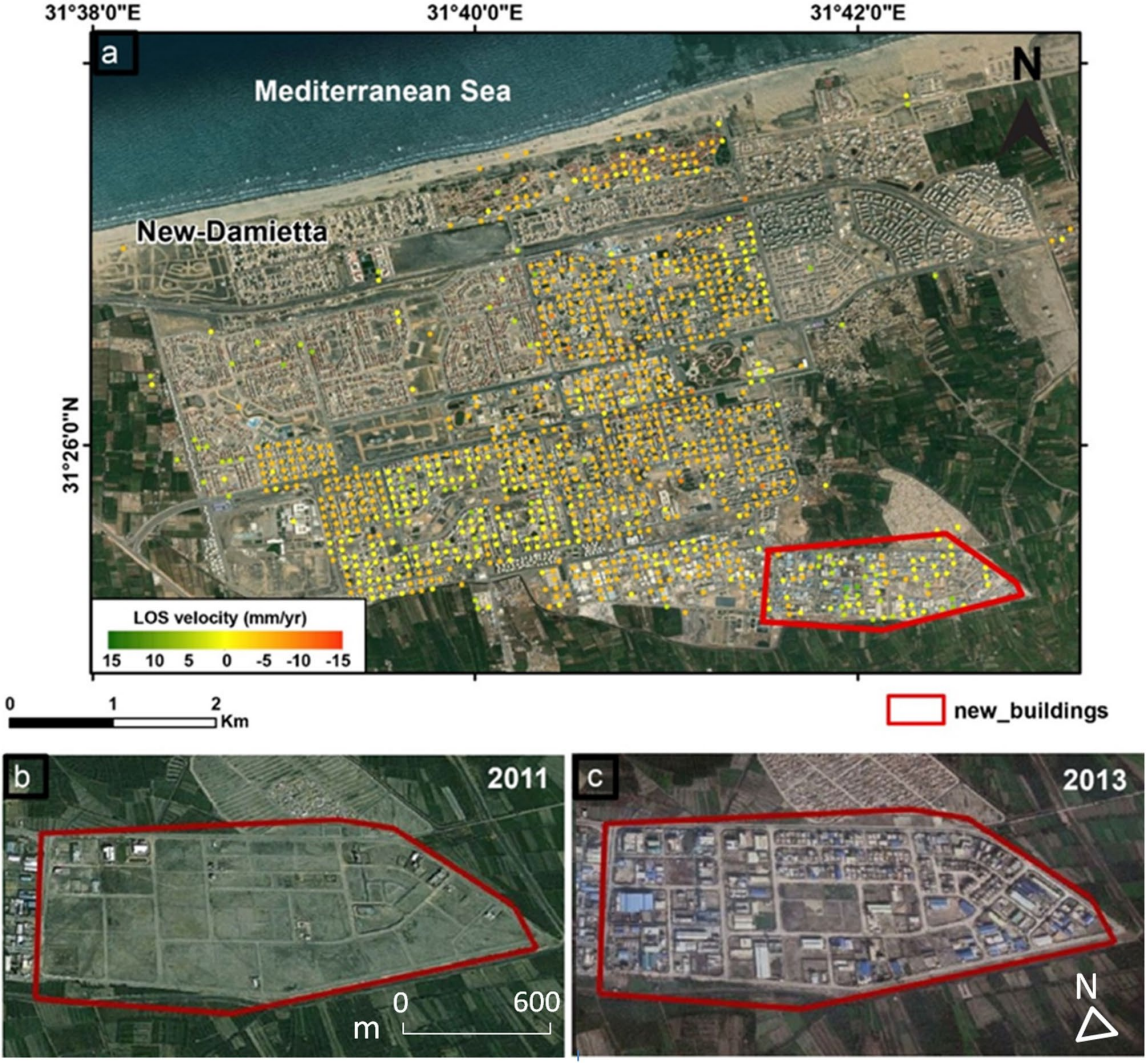
(**a**) The PSI results in New-Damietta city showing moderate subsidence rates from − 3 upto − 5 mm/year, (**b**) Google Earth Satellite image shows a selected area within New-Damietta city devoid of buildings in 2011, (**c**) Google Earth Satellite image shows the new buildings constructed in the same selected region in year 2014. Such excessive constructions led to soil compaction and consequently moderate subsidence rates. The maps were created using ArcGIS, version 10.6.1^76^.

Moving from the broad perspective of the Nile Delta’s deformation pattern to the specific localized deformation that takes place in major cities and villages. As was previously stated at the commencement of this section, our results (Fig. 11) indicate an outstanding correlation between InSAR and GPS data; yet, they also show a significant difference and agreement with previous results. Our findings of the PSI analysis from 2014 to 2019 are showing that the northern eastern parts of the Delta are experiencing the highest rates of subsidence (Damietta (− 11 ± 0.6 mm/year, Mansoura − 8.9 ± 0.6 mm/year and Port Said − 6.3 ± 0.7 mm per year). Such high rates of subsidence are compatible with the results of (1) ^38^ (Damietta − 8 mm/year); (2)^106^ (Mansoura − 9 mm/year); (3) ^36^, the average subsidence rate of the north eastern part − 8.9 mm/year; (4) ^34^ (Damietta − 10.3 mm/year, Mansoura − 10 mm/year and Port Said − 4.9 mm/year); (5 )^41^ (Mansoura − 11 mm/year and Port Said − 7.9 mm/year); and (6) ^48^ (Mansoura − 9.11 mm/year and Port Said − 6.74 mm/year). They were also incompatible with the results of^35^, where Damietta and Port Said subside with low rate approxamtely − 3 to − 5 mm per year. We interpretted such considerable rates of subsidence due to the natural compaction of the thick Quaternary sediments consistent with^38,64^. We can add that urbanisation is a contributing element to subsiding in such region. The subsidence rate in New-Damietta city was showing a moderate one (− 3.8 mm/year), in accordance with^41^, (− 2.7 mm per year), and^48^, (− 3.89 mm per year). The deformation could be explained by the overwhelming burden of urban growth on the un-consolidated sedimetrary succession. Figure 12 is showing the excessive and accelerating growth of urbanization within New-Damietta city, led to such a subsidence. The subsidence due to urbinization is a common phenomenon in the world, such as subsidence in West Pearl River Delta (WPRD)^107^ and Mekong Delta in Vietnam^108^, because of tremendous population growth and rapid urbanization over this region in the last few decades.

The northern western part of the Delta is suffering modest rates of subsidence through our results, where Alexandria city had subsidence rate − 1.6 ± 0.7 mm/year, however its peripheries had greater rates approaching − 5 mm/year. According to^26^, the rate of subsidence in Alexandria was − 0.4 mm/year^35^. , declared that the subsidence rate in Alexandria was between − 3 and − 5 mm/year, but^41^ reported that the rate at Alexandria subsidence was − 6.8 mm/year^109^. reported that the subsidence rate in Alexandria was − 1.3 mm/year. We attributed such steady subsidence to the Holocene sediment thickness’s natural compaction and dewatering, as previously explained by^26,110^. Conversely, the rates were higher around the city’s boundaries because of the sediments’ compaction brought on by urbanization. The noticeable asymmetry between the northern eastern and western portions of the Delta could by interpreted by discrepancies in the age, composition, and thickness of the sediments. Where the northern eastern delta’s Holocene sediments are richer in silt and clay concentration, younger, and thicker. By comparison, their equivalents in the northern western delta are thinner, older and predominantly consist of sand- and clay-sized sediments^38,64^. The cites of Shebin El Kom, Damanhour, Tanta, New-Damietta, Kafr El-Sheikh had moderate subsidence rates (− 3.2 ± 0.6, − 2.4 ± 0.7, − 4.2 ± 0.6, − 3.8 ± 0.7, − 3.2 ± 0.7 mm/year, respectively).

From north to south in the central part of the Nile Delta, we could report the following subsidence rates, Sidi Salem (− 4.1 ± 0.6 mm/year), Kafr el Sheikh (− 3.2 ± 0.7 mm/year), Tanta (− 4.2 ± 0.6 mm/year) and Shebin El Kom (− 3.2 ± 0.6 mm/year). According to^34^, groundwater extraction caused subsidence rates of − 4.0 mm/year in Tanta and − 4.8 mm/year in Mahla. Meanwhile^35^, reported that urbanisation was triggering the central delta region which includes Mit Ghamr, Tanta, Mahala, and Zagazig, to subside at a rate of among − 12 and − 20 mm per year. We think that a combination of anthropogenic factors (groundwater overexploitation and urbanization) might resulted in such subsidence, however we support the idea that Sidi Salem in the northen central part of the Delta might not be influenced by water extraction due to the high salinty of the water in the northern parts of the delta that hindering the procedures of fresh ground water extraction^111^, and further agree with the assertion made by^36^ that it might be impacted by the extraction of gas and oil, which causes the compressible sediments to consolidate and compact as a result of changes in pore pressure and vertical efficacious stress within the reservoir, leading to subsidence^112^. Another odd finding was reported by^41^, who claimed that Tanta (11 mm/year), Damnhour (27.9 mm/year), and Hamoul (1.9 mm/year) stations clearly show signs of uplift in the central Nile Delta. Such rich, diverse, and wide-ranging deformation rates gathered from the Nile Delta demonstrate the delta’s complexity and the necessity for a longer, more thorough investigation, as well as the need for accessibility of a large number of GPS stations for InSAR verification. Table 2 summarises the published subsidence rates of the Nile Delta in comaprison with our estimated rates.

**Table 2:** The previously published rate for the nile Delta in comparison with the rate estimated from this study.

Study	Rates (mm/year)							Technique
	Cairo	Mansoura	Mahala	Port-Said	Tanta	Damietta	Alexandria	
^43^				− 2.5:− 5			-1:-2.5	Field measurements
^113^						− 0.9 : − 4.3		Field measurements
^23^						-4.91	-6.21	Field measurements
^41^		−1.1		−0.79	−1.1		-0.68	Field measurements
^50^	− 5	− 9	− 7					Remote Sensing
^38^						− 4 : -8		Remote Sensing
^34^	− 6.4	− 10.0	− 4.8	− 4.9	− 4.0	− 10.3		Remote Sensing
^106^		−9	−5					Remote Sensing
^114^				-10	-3		-6	Remote Sensing
^26^							−2	Remote Sensing
^115^				-8			-2	Remote Sensing
^36^	−0.4		−9.7				−9.7	Remote Sensing
^35^		−12 to − 20	−12 : −20	−6 : −12	−12 : −20		−3 : −8	Remote Sensing
This Study	-0.1 : -0.2	-8.9 : -9.7	-4 : -5	-6.3 : -6.8	-4 : -6	-3.7 : -3.8	-1.5 : -1.6	Field measurements + Remote Sensing

### Sea level changes along the nile Delta by satellite altimetry

**Fig. 13:**
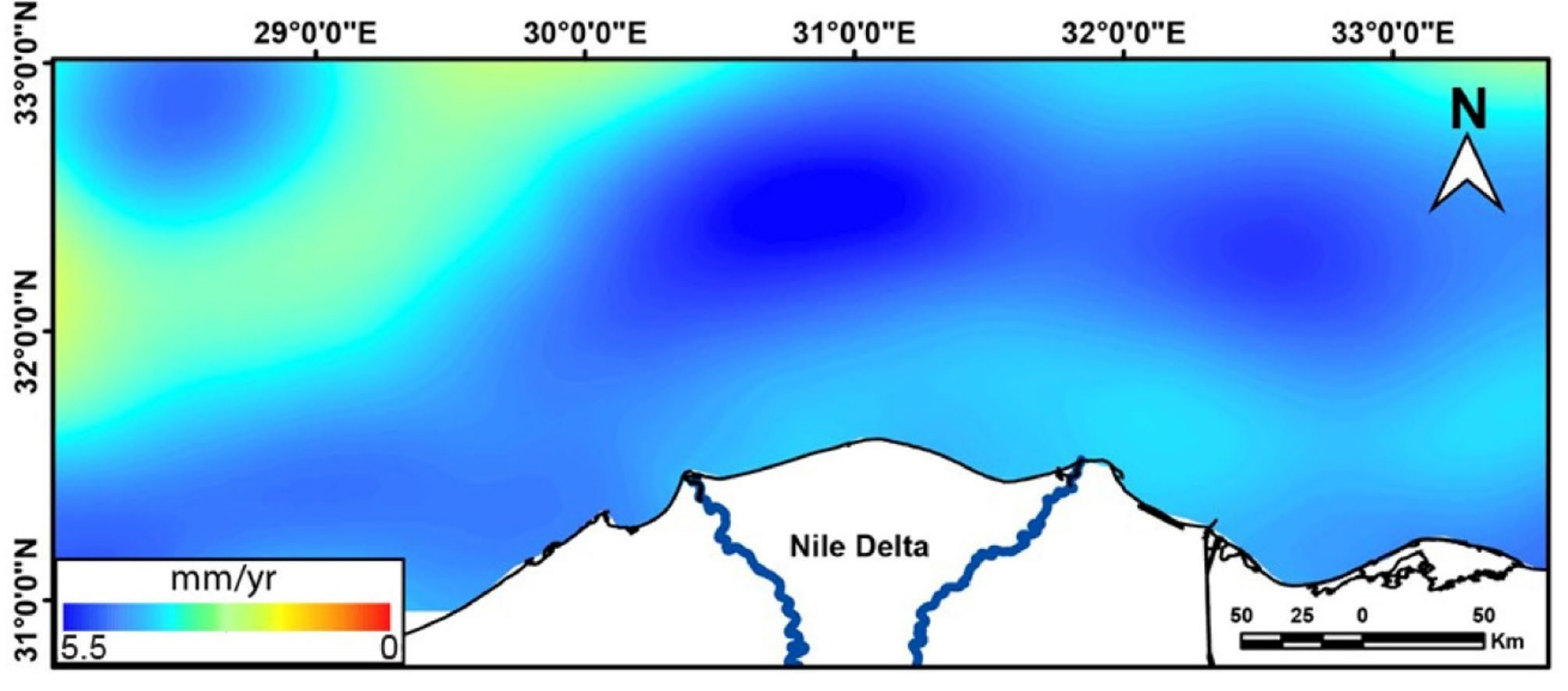
Trend map of SLA along the Nile Delta and surroundings, using altimetric data over the period (1993–2020). The SLA trend was estimated after removing the seasonal cycle and glacial-isostatic adjustment (GIA) correction. The map was created using ArcGIS, version 10.6.1^76^.

In this section, we analyzed in detail the spatiotemporal evolution and the long-term trend of SLA from altimetry over the period from 1993 to 2019. We then estimated the VLM at New-Damietta station by using the difference between altimetry and TG at this station. The spatial distribution of the SLA trend from the altimetry (Fig. 13) indicated a significant (*p* > 0.05) and positive trend over the entire area and ranged between 0.1 and 5.5 mm/year. This trend was estimated after considering the GIA correction of the altimetry data (dGeoid), which was very low in the study areas (between about − 0.05 and − 0.11 mm/year). The maximum SLA trends occurred in the Shikmona and Mersa Matruh eddies, which is consistent with the results of^116^, who found the same SLA trend pattern. The SLA trend over the Nile Delta coast is relatively high at about 3.5 mm/ year, in agreement with^23^. It seems that the sea level rise of the Mediterranean Sea along the central and eastern sides of the Nile Delta is relatively lower (~ 3.5 mm/year) than the western side (~ 4–5 mm/year).

**Fig. 14:**
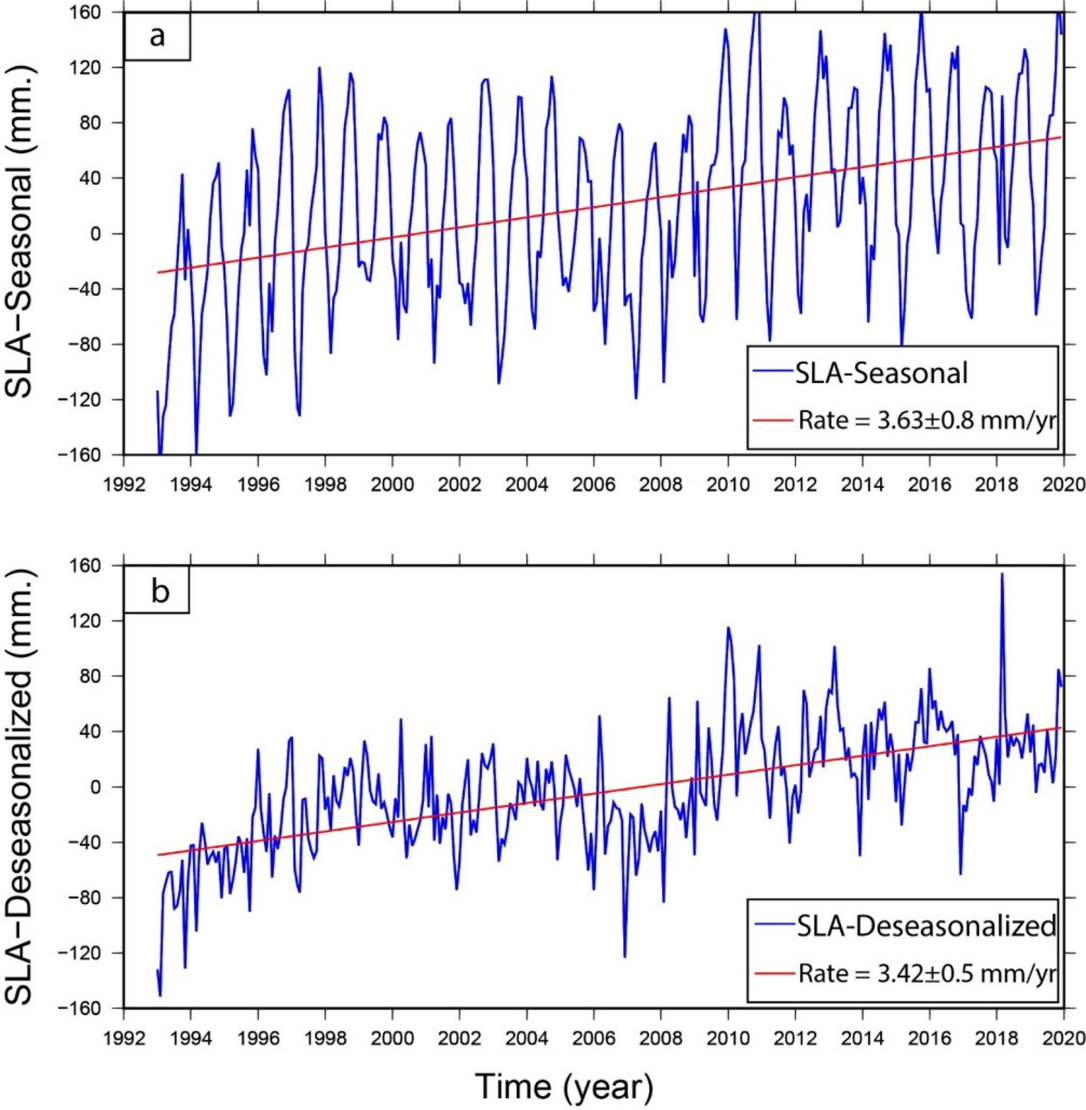
Monthly averaged SLA along the processed region over the Nile Delta from 1993 to 2019. The top panel (**a**) represents the monthly SLA with the seasonal cycle, while the bottom panel (**b**) represents the SLA after removing the seasonal cycle (i.e. the de-seasonalized SLA). The linear trends are shown in the left corners of each panel. Thae figures were created using Generic Mapping Tools (GMT), version 5^19^.

The temporal evaluation and linear trend of the mean SLA among 1993 and 2019 along the southern Mediterranean Sea (the same period as the InSAR and GPS analysis) are shown in Fig. 14a), yielding a trend value of 3.63 ± 0.8 mm/year. After removing the seasonal cycles from the monthly averaged SLA, the trend value became 3.42 ± 0.5 mm/year (Fig. 14b). It seems that the high positive peaks occurred within 2010, 2011, 2013, 2016 and 2018 (Fig. 14b), while the strongest negative peaks occurred in 2007 and 2017.

### Vertical land motion at the Damietta by satellite altimetry and TG

**Fig. 15:**
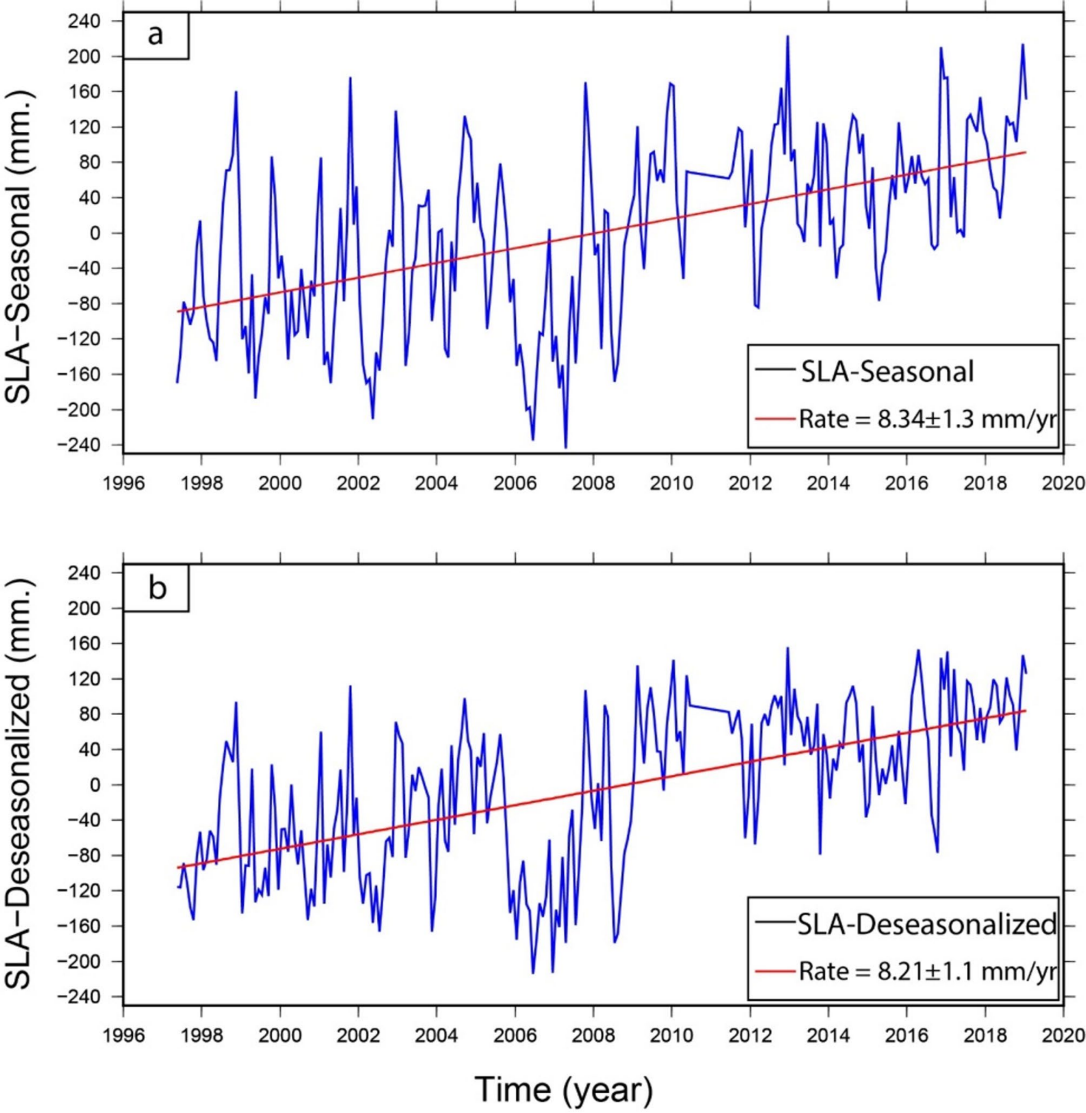
Monthly averaged time series of SLA along the Damietta TG from 1997 to 2019. The top panel (**a**) represents the monthly SLA with the seasonal cycle, while the bottom panel (**b**) represents the SLA after removing the seasonal cycle (i.e. the de-seasonalized SLA). The linear trends are shown in the left corners of each panel. Thae figures were created using Generic Mapping Tools (GMT), version 5^19^.

The tide-gauge station was fixed in New-Damietta city along the shoreline, and data were accessed from 1997 to 2019. The averaged monthly time series for TG gave a trend value of 8.34 ± 1.3 mm/year (Fig. 15a). Once the seasonal cycle was eliminated from the time series, the linear trend of the de-seasoned monthly SLA was about 8.21 ± 1.1 mm/year (Fig. 15b). The value of the TG provides the combination of sea level variation and VLM. The GIA correction at this TG is very small (-0.01 mm/year) and can be negligible.

**Fig. 16:**
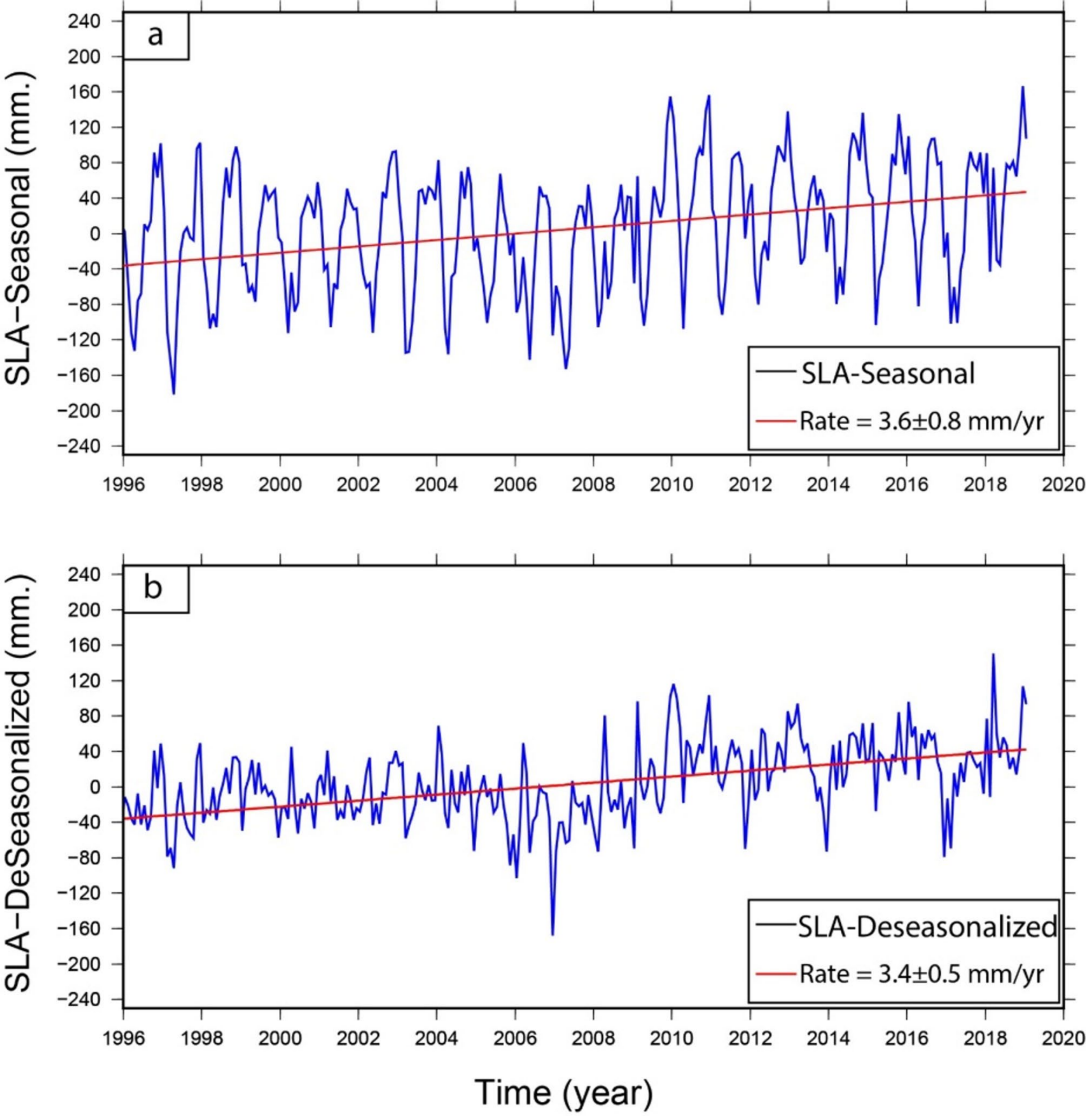
Monthly averaged time series of SLA along the nearest point to Damietta from 1997 to 2019, derived from the processed altimetry data. The top panel (**a**) represents the monthly SLA with the seasonal cycle, while the bottom panel (**b**) represents the SLA after removing the seasonal cycle (i.e. the de-seasonalized SLA). The linear trends are shown in the left corners of each panel. The figures were created using Generic Mapping Tools (GMT), version 5^19^.

The SLA was extracted from the nearest altimetry grid point to New-Damietta TG (Fig. 13) and the SLA trend was calculated along this grid point during the overlap period with the TG (i.e., from 1997 to 2019). The altimetry SLA trend during this overlap period was about 3.8 ± 0.8 and 3.4 ± 0.5 mm/year for the seasonal and de-seasonal SLA, respectively (Fig. 16a, b).

**Fig. 17:**
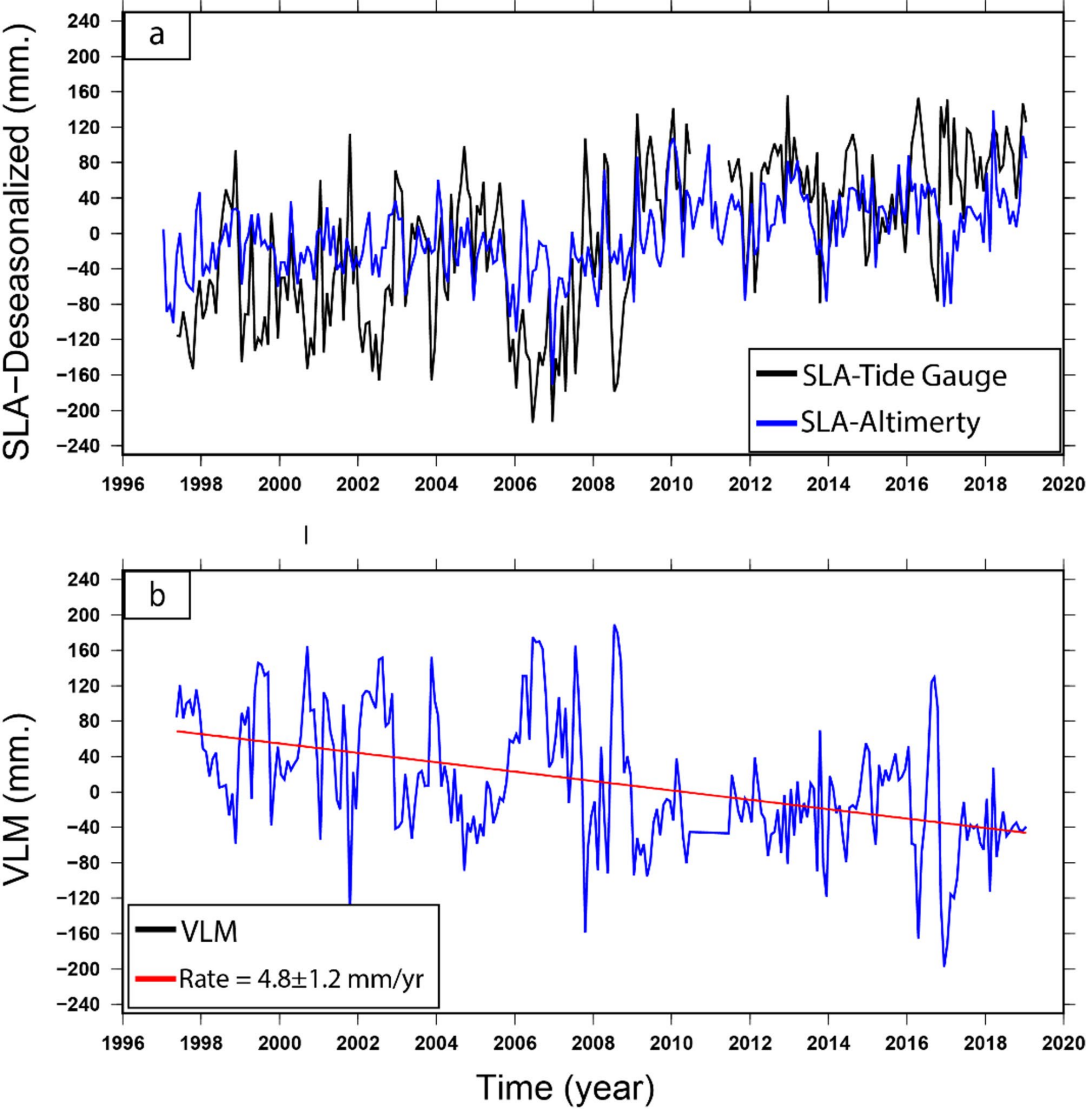
(**a**) Comparison between deseasonalized SLA from tide gauges (black line) and satellite altimetry (blue line) between (1997–2019), showing close pattern correlation. (**b**) VLM within Damietta estimated from (desasonal SLA from Damietta TG minus deseasonal SLA from Altimetry along the nearest point to Damietta TG and giving a value 4.8 ± 1.2 mm/year. Thae map were created using Generic Mapping Tools (GMT), version 5^19^.

The deseasonal SLA trend along Damietta from satellite altimetry is 3.4 ± 0.5 mm/year, while the deseasonal SLA trend from TG along Damietta is 8.21 ± 1.1 mm/year. The difference between these two trend values could be attributed to the VLM. The attitude of their time series was compared in Fig. 17a. The comparisons focus on analyzing the patterns and correlations between the de-seasonal monthly mean SLA at the tide gauge and the nearest altimetry grid point. The correlations and Root Mean Square Differences (RMSD) of the regression between them were selected to measure the goodness of fit^90,98,99^. The observed significant correlation coefficient (0.63) and a low RMSD (7 cm) indicate the good agreement between the TG and the altimetry at this station. The VLM has been estimated by calculating the difference between the de-seasoned SLA from TG and the de-seasoned SLA derived from the time series of the nearest satellite altimetry grid point to the New-Damietta TG. A considerable land subsidence was estimated based on this technique (4.8 ± 1.2 mm/year) (Fig. 17b).

Regarding to the altimetric data, the processing was performed along the grid that encompasses the shoreline of the Nile Delta in the southern eastern region of the Levantine Basin. Despite the coastal altimetry signal being less precise than the open-water altimetry signal, it remains nonetheless valuable, indispensable, and beneficial^117^. The sea level trend was modest along the coastal lines of the Nile Delta (~ 3.5 mm/year), in accordance with^23^. The SLA trend along the central and eastern side of the Nile Delta seemed to be relatively lower (~ 3.5 mm/year) than the western side which experiencing higher rates (~ 4–5 mm/year), meanwhile the results from PSI analysis was showing that the eastern side of the Nile Delta is more subsiding than the western part. Although a correlation between high subsidence and high SLA rates was hypothesised, our study revealed that the relationship in this case was inverse.

According to the inspected long-term sea level change, there were considerable negative peaks in 2007 and 2017, and substantial positive peaks in 2010, 2011, 2013, 2016 and 2018. Localised variations in semi-enclosed basins, for example the Mediterranean Sea, are influenced by global changes in open oceans^29,118^. The Mediterranean Sea may be directly impacted by sea level variance, which is profoundly affected by sea surface temperature, steric sea level and level of sea variation west of the Gibraltar Strait. Furthermore, a change point’s occurrence—whether it be rising or decreasing—may be linked to multiple causes simultaneously. The reported elevated peaks could be correlated with Northern Ionian Gyeare reversal episodes occurrences, which altered the thermohaline characteristics and mass of water redistribution throughout the sub-basins^23,116,119^. Furthermore, a significant negative phase of the North Atlantic Oscillation might have contributed to the negative peaks^23,93^.

A positive aspect through this research is that we were able to calibrate and find significant correlations between the results of various approaches along the New-Damietta city. This approach has been implemented in various past studies^105^. The results of VLM at New-Damietta city deduced from PSI, GPS, and the subtraction of altimetry and TG were calibrated and gave a VLM of *−* 3.8 ± 0.6 mm per year, *−* 3.7 ± 1.4 mm per year, and *−* 4.8 ± 1.2 mm/year, correspondingly (Table 3).

**Table 3:** VLM rates according to the difference between de-seasoned SLA from satellite altimetry and TG within Damietta City, in addition PSI and GPS.

Tide gauge SLA (mm/year)		Satellite SLA (mm/year)		VLM rate (mm/year) TG minus Altimetry	PSI-VLM rate (mm/year)	GPS-VLM rate(mm/year)
Seasonal	De-seasonal	Seasonal	De-Seasonal	–	–	–
8.34 ± 1.3	8.21 ± 1.1	3.6 ± 0.8	3.4 ± 0.5	− 4.8 ± 1.2	− 3.8 ± 0.6	− 3.7 ± 1.4

### Risk assessment inundation of nile Delta by GIS

**Fig. 18:**
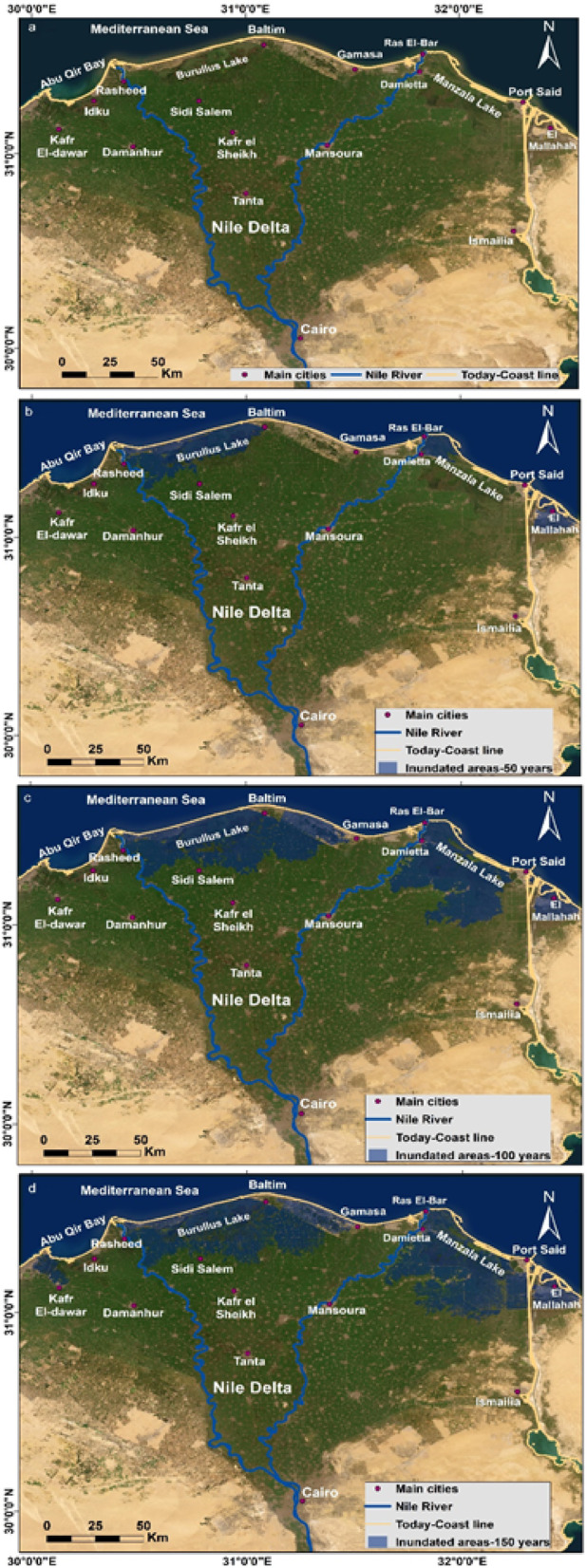
Inundation model of the Nile Delta by Mediterranean Sea encroachment, (**a**) the current situation, (**b**) the scenario after 50 years, (**c**) the scenario after 100 years, and (**d**) the scenario after 150 years. The map was created using ArcGIS, version 10.6.1^76^.

The simulation model of innudation created in this study incorporated the results of PSI rates along the Nile Delta region and GNSS velocities from the studied stations, and employed high spatial and vertical resolution digital elevation model TanDEM-X {resolution: 12 m, relative horizontal accuracy: 3 m, and relative vertical accuracy: two meter (slope ≤ 20%), four meter (slope ≥ 20%)}. Besides, a linear rate of SLA 3.42 mm/year was assumed for 50 years, 100 years and 150 years. It is worth noting that the uncertainties of the inputs to the simulation model were taken into account. Also, this simulation is based on our newly estimated rates (subsidence, SLR, topography ect.) and in case these rates changed, the simulation model may change too. The results indicated that the current rate of SLR isn’t a potential threat to the Nile Delta (Fig. 18a). The simulation by 50 years is showing inundation for the entire Burullus western of the delta, eastern of the Nile Delta (zone west of Damietta and Ras EL-Bar and El Mallahah west of Port Said). The inundated segments through this scenario is ~ 482 km^2^ (Fig. 18b). In case of 100 years simulation (Fig. 18c), we could see invasion of ~ 2433 km^2^, covering the entire Burullus area, and reaching south near the northern borders of Sidi Salem and west reaching Rasheed promentory, besides the area between Baltim and Gamasa, in addition Manzala Lake extending south covering some villages, and El Mallahah west of Port Said. And finally the worst-case 150 years scenario, invaded area ~ 3320 km^2^, (Fig. 18d). The inundated zones resembled the pattern of the previous scenario (100 years) with more extension, and invasion of west of Abu Qir Bay. Large segments of the Nile Delta could be submerged under these scenarios. It was noticable that the areas of Baltim and Ras El-Bar were protected from invasion throughout the different scenarios.

We assessed the impact of SLR on coastal regions after 50, 100 and 150 years by simulating sea encroachment and creating some scenarios (Fig. 18). Such task was accomplished using the results of subsidence rates from InSAR and GNSS, and the estimated SLA from altimetry, and high resolution DEM (TerraSAR-X/TanDEM- X). Few inundation models took into consideration the land subsidence factor in their evaluation based on geodetic measurements and/or satellite missions as InSAR, e.g^36^. , . In fact, most previous inundation models did not incorporate all the factors we have used. Even in^36^, while they accounted for subsidence, they did not calculate SLR themselves but instead applied a value from the IPCC. The land percentages that could be affected or flooded in the northern Mediterranean coast of the Nile Delta region were variable. Some comparisons between different models would be represented as follows; (1) our results revealed that wide areas of the northern Delta could be affected by steady SLR of 3.42 mm per year and continuing land subsidence. Almost ~ 482 km^2^ will be affected by 50 years at worst scenarios, ~ 2433 km^2^ will be affected by 100 years, and ~ 3320 km^2^ over 150 years at worst scenarios, which means large segments of the northern Nile Delta could be submerged; (2) the inundated areas of the delta that estimated from the SLR in 2050 model were about 50 km^2^. The SLR rates used in that study were from only 2 TG stations, one in Alexandria and the other in Port Said with an average rate of 0.6 mm per year^8^; (3) an estimated 2,660 km^2^ in northern delta could be inundated by year 2100 with eustatic SLR of 0.44 m^36^; (4) the residential and agricultural areas could be flooded by the SLR of 0.5 m are 975 km^2^^52^; (5) under the conditions of a sea level rise of 0.5 millimeters per year, roughly 37.96% of the total area of the Nile Delta Coastal Governorates would be susceptible to flooding by the year 2100 on average^53^; and (6) about 1800 km^2^ of cropland, wetland and fish ponds, representing 7.5% of the total delta area (23,850.76 km^2^), could be submerged in case of 0.5 m SLR^120^.

Throughout the different conducted scenarios (Fig. 17b, c,d), it was visible that Ras El-Bar and Baltim were shielded from attack by SLR. It is worth mentioning that a section of the east-west trending sand dune field protects the lands north of Baltim from sea water invasion (width: more than five kilometers, length: twenty five kilometers; minimum elevation: five meter)^121^. Moreover, the western part of RAS El-Bar till Damietta harbor also is defended by sea wall extending more than 2 m in height^122^.

**Fig. 19:**
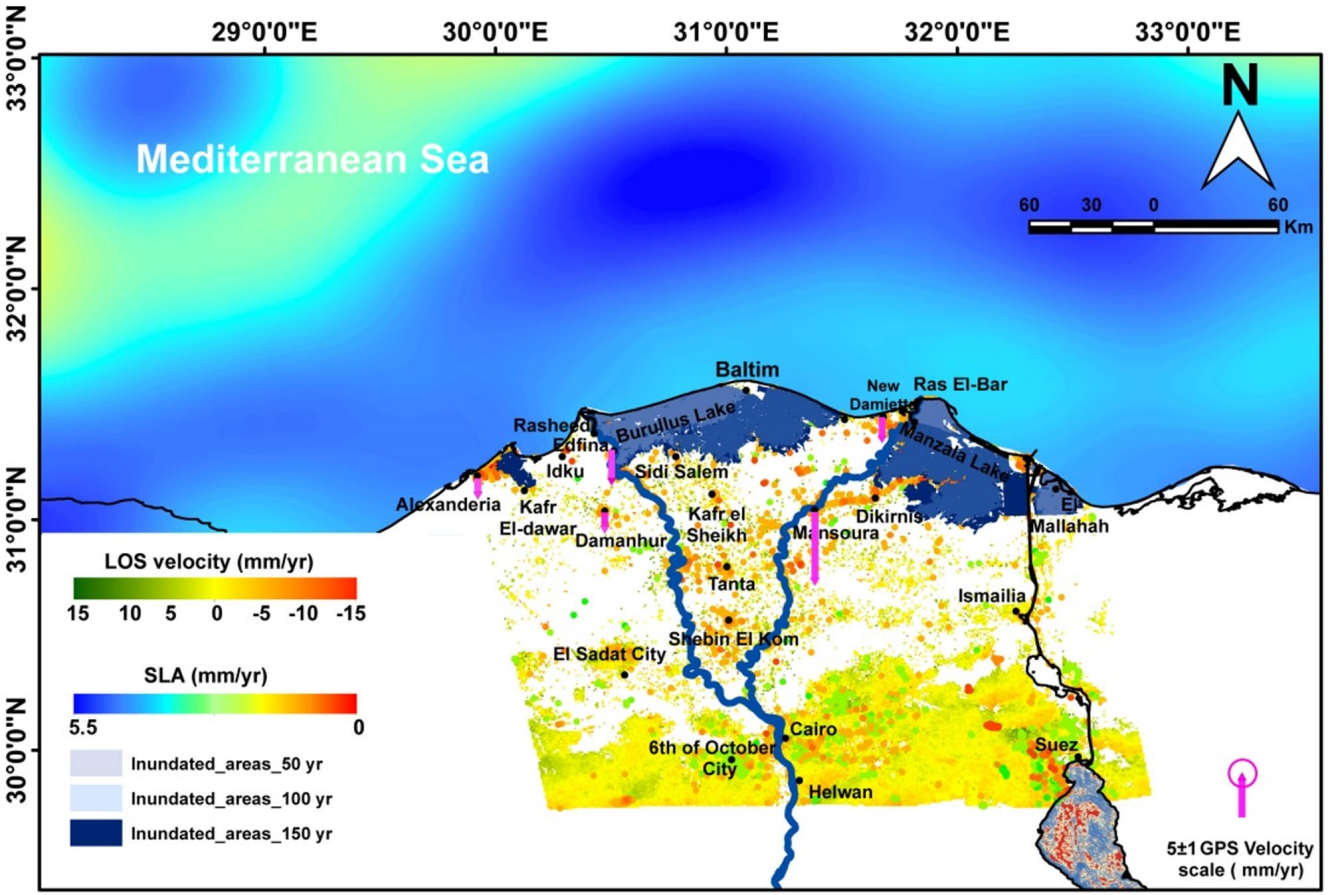
Final intergrated model of the Nile Delta showing all the approaches (PSI, GNSS, altimetry) employed through the study, and ultimately contributed in creation the inundation scenarios over 50, 100 and 150 years. The map was created using ArcGIS, version 10.6.1^76^.

Our research represents a comprehensive approach for assessing the deformation of the Nile Delta and the Mediterranean Sea rise, ultimately generating various scenarios of encroachment and inundation (Fig. 19).

Our research findings reveals a predominately human control (anthropogenic effects) over the land deformation, along with a localized subsidence in the major cities, due to the nature of the sedimentary succesion covering the Delta. Besides, the Mediterranean SLR could represent a fatal disaster based on plausible futures conducted through the study. Given these facts, the decision makers ought to consider such scenarios that pose significant potential risks. Among the hazards are the possibility of forcing the residents of coastal zones to leave their communities or, at the least, relocate some of them south; the destruction of coastal resorts and large portions of agricultural are other challenges. Therefore, in order to secure the Nile Delta, the government needs to come up with innovative solutions and a sustainable management plan.

## Limitations and uncertainties

The list of factors below may affect the accuracy of the deformation rates utilizing PSI techniques as well as the inundation scenarios: (1) The dense vegetation in the Nile Delta causes extremely low coherence, which in turn leads to the production of interferograms of poor quality that provide less precise deformation rates. The accuracy of the sea encroachment simulation model is directly influenced by the quality of the PSI deformation rates., (2) Uncertainty in such scenarios may also result from the selected method of interpolation of the extracted individual PS deformation, in order to fill the gaps in areas with no coherent PS identified; (3) The hypothesis of steady SLR (3.4 mm/year) along the entire shore line of northern Egypt is not fully true, that may lead to exaggeration or under-estimation in the simulation model; (4) When the sea level rise projections do not account for different RCP scenarios, it represents a considerable limitation, as it fails to capture the range of possible future outcomes depending on global emissions pathways. Integrating multiple RCP scenarios would provide a more comprehensive understanding of how sea level rise could evolve over time, helping to better inform flood risk assessments and long-term planning; (5) Additionally, the possibility of subsidence rates changing over time should be considered, as subsidence can interact with rising sea levels to exacerbate flooding. Accounting for both RCP-driven sea level rise and potential changes in subsidence rates would enhance the robustness and accuracy of the flood risk model; and (6) Lack of GPS and TG stations led to less assessment and calibration of the study, however, this study is considered a rich one than others, with significant number of GPS stations beside interferometry technique. In addition, we initiated considerable number of those stations (GPS and TG) through our project funded from Academy of Scientific Research and Technology (ASRT) in Egypt. The coming research will involve wider time span of studying interferometry and more GPS and TG stations.

## Conclusions

Nile Delta subsidence and Mediterranean Sea encroachment to the Delta represent challenges to the Egyptian government. In this study, we investigated present rates and patterns of ground deformation in Nile Delta using Sentinel-1 data ascending scenes spanning a duration of five years (2014 to 2019) and applying the PSI technique. Measured subsidence rates vary widely across the Nile Delta, and concentrated in major cities and subjected mainly to anthropogenic reasons, besides some natural factors. The rates could be summarized as the following. (1) major cities and urban areas adjacent to the two primary active branches of the Nile Delta (Rosetta and Damietta), have experienced the highest rates of subsidence; (2) specifically, the cities of Damietta, Mansoura, and Port Said on the eastern side of the Nile Delta have recorded the highest rates of subsidence, with values of -11 ± 0.6, -8.9 ± 0.7, and − 6.3 ± 0.7 mm/year, respectively, such subsidence could be attributed to the natural compaction of the thick Quaternary sediments consistent, along with the effects of urbanization, particularly in the peripheries of cities; (3) moderate subsidence rates have been observed in cities such as Shebin El Kom (-3.2 ± 0.6) due to groundwater overexploitation and urbanization, Damanhour (-2.4 ± 0.7) groundwater overexploitation and urbanization, Tanta (-4.2 ± 0.6),, New-Damietta (-3.8 ± 0.6) due to urbanization growth, Kafr El-Sheikh (-3.2 ± 0.7) due to groundwater overexploitation and urbanization, and Sidi Salem (-4 ± 0.6) due to extraction of gas and oil, which causes the compressible sediments to consolidate and compact as a result of changes in pore pressure and vertical efficacious stress within the reservoir, leading to subsidence, and (5) eight stations of GPS were analyzed to calculate the deformation rate and showed compitability with the results of PSI.

Satellite altimetry along the Mediterranean coast and TG along New-Damietta city were corrected for atmospheric errors and de-seasonal anomalies were calculated. This gave a fair picture about sea level attitude. The linear trend of SLA from satellite altimetry had given a value 3.42 mm/year. As well, the difference between the de-seasoned SLA from the TG and the de-seasoned obtained from the time series of the closest satellite altimetry grid to the New-Damietta TG was used to estimate the VLM. This methodology yielded a considerable land subsidence estimate (-4.8 mm/year). The results were then compared with a single GPS station, which recorded a VLM of -3.7 mm/year, and with the nearest PSI points, which showed a VLM of -3.8 mm/year.

Furthermore, using a combination of deformation estimates from the PSI analysis, GPS, a high-resolution digital elevation model, predicted SLR values, the regions susceptible to sea encroachment by 50, 100 and 150 years were identified. SLR of 3.4 mm/year and continued land subsidence will damage large parts of the northern Delta. Large areas of the northern Nile Delta are predicted to flood in the worst-case scenarios, which involve impacts of about 482 km^2^ over 50 years, 2433 km^2^ over 100 years, and 3320 km^2^ over 150 years. The decesion-makers should pay attention to such scenarios for mitigating the potential threats to the Nile Delta.

To mitigate these risks and protect vulnerable areas of the Nile Delta, **decision-makers** should consider the following strategies:

1. **Establish a continuous GNSS monitoring network** to track subsidence trends more accurately and in real-time.
2. **Implement groundwater extraction regulations** to minimize anthropogenic subsidence, particularly in high-risk urban areas.
3. **Develop sustainable coastal protection measures**, such as seawalls and artificial sand nourishment, to combat Mediterranean Sea encroachment.
4. **Enhance urban planning policies** to restrict construction in high-risk subsiding areas and promote adaptive infrastructure.
5. **Promote international collaboration** to integrate advanced satellite monitoring techniques (e.g., InSAR, GNSS) into long-term land deformation studies.
6. **Increase public awareness and preparedness efforts** to ensure communities understand the potential risks and necessary mitigation measures.

## Data Availability

The datasets generated and/or analyzed during the current study (InSAR and Altimetry) are available in the Alaskan Satellite Facility (ASF) and the Copernicus Marine Environment Monitoring Service (CMEMS), respectively. Meanwhile, The datasets of GNSS and TG analyzed during the current study are not publicly available due to internal regulations, but are available from the corresponding author on reasonable request.
